# Integrals for functions with values in a partially ordered vector space

**DOI:** 10.1007/s11117-015-0392-y

**Published:** 2015-12-30

**Authors:** A. C. M. van Rooij, W. B. van Zuijlen

**Affiliations:** 1 Department of Mathematics, Radboud University Nijmegen, P.O. Box 9010, 6500 GL Nijmegen, The Netherlands; 2 Mathematical Institute, Leiden University, P.O. Box 9512, 2300 RA Leiden, The Netherlands

**Keywords:** Partially ordered vector space, Riesz space, Bochner integral, Pettis integral, Integral, Vertical extension, Lateral extension, 28B05, 28B15

## Abstract

We consider integration of functions with values in a partially ordered vector space, and two notions of extension of the space of integrable functions. Applying both extensions to the space of real valued simple functions on a measure space leads to the classical space of integrable functions.

## Introduction

For functions with values in a Banach space there exist several notions of integration. The best known are the Bochner and Pettis integrals (see [1, 2]). These have been thoroughly studied, yielding a substantial theory (see Chapter III in the book by Hille and Phillips [3]).

As far as we know, there is no notion of integration for functions with values in a partially ordered vector space; not necessarily a $\sigma$-Dedekind complete Riesz space. In this paper we present such a notion. The basic idea is the following. (Here, *E* is a partially ordered vector space in which our integrals take their values.)

In the style of Daniell [4] and Bourbaki [5, Chapter 3,4], we do not start from a measure space but from a set *X*, a collection $\Gamma$ of functions $X\rightarrow E$, and a functional $\varphi : \Gamma \rightarrow E$, our “elementary integral”. We describe two procedures for extending $\varphi$ to a larger class of functions $X\rightarrow E$. The first (see Sect. 3), the “vertical extension”, is analogous to the usual construction of the Riemann integral, proceeding from the space of simple functions. The second (see Sect. 4), the “lateral extension”, is related to the improper Riemann integral.

In Sect. 5 we investigate what happens if one repeatedly applies those extension procedures, without considering the space *E* to be $\sigma$-Dedekind complete or even Archimedean. However, under some mild conditions on *E* one can embed *E* into a $\sigma$-Dedekind complete space. In Sect. 6 we discuss the extensions procedures in the larger space. Sections 7 and 8 treat the situation in which $\Gamma$ consists of the simple *E*-valued functions on a measure space. (In Sect. 7 we have $E=\mathbb {R}$.) In Sect. 9 we consider connections of our extensions with the Bochner and the Pettis integrals for the case where *E* is a Banach lattice. In Sect. 10 we apply our extensions to the Bochner integral. For more alternative approaches we refer to the discussion in Sect. 11.

## Some notation

$$\mathbb {N}$$ is $$\{1,2,3,\ldots \}$$.

Let *X* be a set. We write $\mathcal {P}(X)$ for the set of subsets of *X*. For a subset *A* of *X*:

$$\begin{aligned} \mathbbm {1}_A(x) = {\left\{ \begin{array}{ll} 1 &{} \text{ if } x\in A, \\ 0 &{} \text{ if } x\notin A. \end{array}\right. } \end{aligned}$$

As a shorthand notation we write $\mathbbm {1}= \mathbbm {1}_X$.

Let *E* be a vector space. We write $x= (x_1,x_2,\ldots )$ for functions $x: \mathbb {N}\rightarrow E$ (i.e., elements of $E^\mathbb {N}$) and we define

$$\begin{aligned} c_{00}[E]&= \{ x\in E^\mathbb {N}: \exists N \ \forall n\ge N \left[ x_n =0 \right] \}, \quad c_{00} = c_{00}[\mathbb {R}] \end{aligned}$$

We write $c_0$ for the set of sequences in $\mathbb {R}$ that converge to 0, *c* for the set of convergent sequences in $\mathbb {R}$, $\ell ^\infty (X)$ for the set of bounded functions $X\rightarrow \mathbb {R}$, $\ell ^\infty$ for $\ell ^\infty (\mathbb {N})$, and $\ell ^1$ for the set of absolutely summable sequences in $\mathbb {R}$. We write $e_n$ for the element $\mathbbm {1}_{\{n\}}$ of $\mathbb {R}^\mathbb {N}$.

For a complete $\sigma$-finite measure space $(X,\mathcal {A},\mu )$ we write $\mathcal {L}^1(\mu )$ for the space of integrable functions, $L^1(\mu )= \mathcal {L}^1(\mu )/\mathcal {N}$ where $\mathcal {N}$ denotes the space of functions that are zero $\mu$-a.e. Moreover we write $L^\infty (\mu )$ for the space of equivalence classes of measurable functions that are bounded almost everywhere.

For a subset $\Gamma$ of a partially ordered vector space $\Omega$, we write $\Gamma ^{+}=\{f\in \Gamma : f \ge 0\}$. If $\Lambda , \Upsilon \subset \Omega$ and $f\le g$ for all $f\in \Lambda$ and $g\in \Upsilon$ we write $\Lambda \le \Upsilon$; if $\Lambda =\{f\}$ we write $f \le \Upsilon$ instead of $\{f\} \le \Upsilon$ etc. For a sequence $(h_n)_{n\in \mathbb {N}}$ in a partially ordered vector space we write $h_n \downarrow 0$ if $h_1 \ge h_2 \ge h_3 \ge \cdots$ and $\inf _{n\in \mathbb {N}}h_n=0$.

## The vertical extension

**Throughout this section,** $\mathbf{E}$ **and** $\varvec{\Omega }$ **are partially ordered vector spaces**, $\varvec{\Gamma } \varvec{\subset } \varvec{\Omega }$ **is a linear subspace and** $\varvec{\varphi :} \varvec{\Gamma } \varvec{\rightarrow } \mathbf{E}$ **is order preserving and linear. Additional assumptions are given in** 3.14.

### **Definition 3.1**

Define

1 $$\begin{aligned} \Gamma _v&= \Big \{ f\in \Omega : \sup _{\sigma \in \Gamma : \sigma \le f } \varphi (\sigma ) = \inf _{ \tau \in \Gamma : \tau \ge f} \varphi (\tau ) \Big \}, \end{aligned}$$

and $$\varphi _v : \Gamma _v \rightarrow E$$ by

2 $$\begin{aligned} \varphi _v (f) = \sup _{\sigma \in \Gamma : \sigma \le f } \varphi (\sigma ) \quad (f\in \Gamma _v). \end{aligned}$$

Note: If $f\in \Omega$ and there exist subsets $\Lambda , \Upsilon \subset \Gamma$ with $\Lambda \le f \le \Upsilon$ such that $\sup \varphi (\Lambda ) = \inf \varphi (\Upsilon )$, then $f\in \Gamma _v$ and $\varphi _v (f) =\inf \varphi ( \Upsilon )$.

### **3.2**

The following observations are elementary.

- $\Gamma \subset \Gamma _v$ and $\varphi _v(\tau ) = \varphi (\tau )$ for all $\tau \in \Gamma$.
- $\Gamma _v$ is a partially ordered vector space and $\varphi _v$ is a linear order preserving map^1^.
- $$(\Gamma _v)_v = \Gamma _v$$ and $$(\varphi _v)_v= \varphi _v$$.
- If $\Pi$ is a subset of $\Gamma$, then $\Pi _v \subset \Gamma _v$.

Of more importance to us than $\Gamma _v$ and $\varphi _v$ is the following variation in which we consider only countable subsets of $\Gamma$.

### **Definition 3.3**

Let $\Gamma _V$ be the set consisting of those *f* in $\Omega$ for which there exist countable sets $\Lambda , \Upsilon \subset \Gamma$ with $\Lambda \le f \le \Upsilon$ such that

3 $$\begin{aligned} \sup \varphi (\Lambda ) = \inf \varphi (\Upsilon ). \end{aligned}$$

From the remark following Definition 3.1 it follows that $\Gamma _V$ is a subset of $\Gamma _v$ and that (for *f* and $\Lambda$ as above) $\varphi _v(f)$ is equal to $\sup \varphi (\Lambda )$. We will write $\varphi _V=\varphi _{v}|_{\Gamma _V}$. We call $\Gamma _V$ the *vertical extension* 2 under $\varphi$ of $\Gamma$ and $\varphi _V$ the *vertical extension* of $\varphi$.

In what follows we will only consider $\varphi _V$ and not $\varphi _v$. However, most of the theory presented can be developed similarly for $\varphi _v$. (For comments see 11.2.)

### *Example 3.4*

$\Gamma _V$ is the set of Riemann integrable functions on [0, 1] and $\varphi _V$ is the Riemann integral in case $E=\mathbb {R}$, $\Omega = \mathbb {R}^{[0,1]}$ and $\Gamma$ is the linear span of $\{\mathbbm {1}_{I}: I \text{ is } \text{ an } \text{ interval } \text{ in } [0,1]\}$ and $\varphi$ is the Riemann integral on $\Gamma$.

### **3.5**

In analogy with 3.2 we have the following.

- $\Gamma \subset \Gamma _V$ and $\varphi _V(\tau ) = \varphi (\tau )$ for all $\tau \in \Gamma$.
- $\Gamma _V$ is a partially ordered vector space and $\varphi _V$ is a linear order preserving map.
- $$(\Gamma _V)_V = \Gamma _V$$ and $$(\varphi _V)_V= \varphi _V$$.
- If $$\Pi \subset \Gamma $$, then $$\Pi _V \subset \Gamma _V$$.

### **Definition 3.6**

Let *D* be a linear subspace of *E*. *D* is called *mediated* in *E* if the following is true:

4 $$\begin{aligned}&\text{ If }~A~\text{ and }~B~\text{ are } \text{ countable } \text{ subsets } \text{ of } D~\text{ such } \text{ that }~\inf A-B=0~\text{ in }~E,~\text{ then } \nonumber \\&A~ \text{ has } \text{ an } \text{ infimum } \text{(and } \text{ consequently }~ B~\text{ has } \text{ a } \text{ supremum } \text{ and }~ \inf A = \sup B). \end{aligned}$$

*D* is mediated in *E* if and only if the following requirement (equivalent with order completeness in the sense of [6], for $D=E$) is satisfied

5 $$\begin{aligned}&\text{ If }~A~\text{ and }~B~\text{ are } \text{ countable } \text{ subsets } \text{ of }~D~\text{ such } \text{ that } \inf A-B=0~\text{ in }~E, ~\text{ then } \nonumber \\&\text{ there } \text{ exists } \text{ an }~h\in E~\text{ with }~B \le h\le A. \end{aligned}$$

We say that *E* is *mediated* if *E* is mediated in itself.

Note: if *D* is mediated in *E*, then so is every linear subspace of *D*. Every $\sigma$-Dedekind complete *E* is mediated, but so is $\mathbb {R}^2$, ordered lexicographically. Also, $c_{00}$ and $c_0$ are mediated in *c*, but *c* is not mediated.

With this the following lemma is a tautology.

### **Lemma 3.7**

Suppose $\varphi (\Gamma )$ is mediated in *E*. Let $f\in \Omega$. Then $f\in \Gamma _V$ if and only if there exist countable sets $\Lambda , \Upsilon \subset \Gamma$ with $\Lambda \le f \le \Upsilon$ such that

6 $$\begin{aligned} \inf _{\tau \in \Upsilon , \sigma \in \Lambda } \varphi (\tau - \sigma ) =0. \end{aligned}$$

The next example shows that $\Gamma _V$ is not necessarily a Riesz space even if *E* and $\Gamma$ are. However, see Corollary 3.10.

### *Example 3.8*

Consider $E=c$, $\Gamma = c\times c$, $\Omega = \ell ^\infty \times \ell ^\infty$. Let $\varphi : \Gamma \rightarrow c$ be given by $\varphi (f,g) = f+g$. For all $f\in \ell ^\infty$ there are $h_1,h_2,\ldots \in c$ with $h_n \downarrow f$. It follows that, $\Gamma _V = \{(f,g)\in \ell ^\infty \times \ell ^\infty : f + g \in c\}$. Note that $\Gamma _V$ is not a Riesz space since for every $f\in \ell ^\infty$ with $f\ge 0$ and $f\notin c$ we have $(f,-f)\in \Gamma _V$ but $(f,-f)^{+}= (f,0)\notin \Gamma _V$.

### **Lemma 3.9**

Suppose $\varphi (\Gamma )$ is mediated in *E*. Let $\Theta : \Omega \rightarrow \Omega$ be an order preserving map with the properties:

- if $\sigma , \tau \in \Gamma$ and $\sigma \le \tau$, then $0 \le \Theta (\tau ) - \Theta (\sigma ) \le \tau - \sigma$;
- $$\Theta (\Gamma ) \subset \Gamma _V$$.

Then $$\Theta (\Gamma _V) \subset \Gamma _V$$.

### *Proof*

Let $f\in \Gamma _V$ and let $\Lambda , \Upsilon \subset \Gamma$ be countable sets with $\Lambda \le f \le \Upsilon$ satisfying (6). Then $\Theta (\Lambda ) \le \Theta (f) \le \Theta (\Upsilon )$ and

7 $$\begin{aligned} \inf _{\tau \in \Theta (\Upsilon ), \sigma \in \Theta (\Lambda )} \varphi ( \tau - \sigma ) = \inf _{\tau \in \Upsilon , \sigma \in \Lambda } \varphi \big ( \Theta (\tau ) - \Theta ( \sigma ) \big ) \le \inf _{\tau \in \Upsilon , \sigma \in \Lambda } \varphi ( \tau - \sigma ) =0. \end{aligned}$$

$$\square $$

### **Corollary 3.10**

Suppose that $\varphi (\Gamma )$ is mediated in *E*. Suppose $\Omega$ is a Riesz space and $\Gamma$ is a Riesz subspace of $\Omega$. Then so is $\Gamma _V$.

### *Proof*

Apply Theorem 3.9 with $\Theta (\omega ) = \omega ^{+}$. $\square$

### **3.11**

If $\Gamma$ is a directed set, i.e., $\Gamma = \Gamma ^{+} - \Gamma ^{+}$, then so is $\Gamma _V$. Indeed, if $f\in \Gamma _V$, then there exist $\sigma , \tau \in \Gamma ^{+}$ such that $f \ge \tau - \sigma$ and thus $f = ( f + \sigma ) - \sigma \in \Gamma _V^{+} - \Gamma _V^{+}$.

### **3.12**

In the last part of this section we will consider a situation in which $\Omega$ has some extra structure. But first we briefly consider the case where *E* is a Banach lattice with $\sigma$-order continuous norm. As it turns out, such an *E* is mediated (see Theorem 4.24), but is not necessarily $\sigma$-Dedekind complete (consider the Banach lattice *C*(*X*) where *X* is the one-point compactification of an uncountable discrete space). For such *E* we describe $\Gamma _V$ in terms of the norm.

### **Theorem 3.13**

Let *E* be a Banach lattice with a $\sigma$-order continuous norm. Let $\Omega$ be a Riesz space and $\Gamma$ be a Riesz subspace of $\Omega$. For $f\in \Omega$ we have: $f\in \Gamma _V$ if and only if for every $\varepsilon >0$ there exist $\sigma , \tau \in \Gamma$ with $\sigma \le f \le \tau$ and $\Vert \varphi (\tau ) - \varphi (\sigma ) \Vert <\varepsilon$.

### *Proof*

First, assume $f\in \Gamma _V$. As $\Gamma$ is a Riesz subspace of $\Omega$ there exist sequences $(\sigma _n)_{n\in \mathbb {N}}$ and $(\tau _n)_{n\in \mathbb {N}}$ in $\Gamma$ such that $\sigma _n \uparrow$, $\tau _n \downarrow$,

8 $$\begin{aligned} \sigma _n \le f \le \tau _n \quad (n\in \mathbb {N}), \quad \sup _{n\in \mathbb {N}}\varphi (\sigma _n )= \inf _{n\in \mathbb {N}}\varphi (\tau _n). \end{aligned}$$

Then $\varphi (\tau _n - \sigma _n) \downarrow 0$ in *E*, so $\Vert \varphi (\tau _n)-\varphi (\sigma _n)\Vert \downarrow 0$ and we are done.

The converse: For each $n\in \mathbb {N}$, choose $\sigma _n,\tau _n\in \Gamma$ for which

9 $$\begin{aligned} \sigma _n \le f \le \tau _n, \quad \Vert \varphi (\tau _n) - \varphi (\sigma _n)\Vert \le n^{-1}. \end{aligned}$$

Setting $\sigma _n' = \sigma _1 \vee \cdots \vee \sigma _n$ and $\tau _n'= \tau _1 \wedge \cdots \wedge \tau _n$ we have, for each $n\in \mathbb {N}$

10 $$\begin{aligned} \sigma _n', \tau _n'\in \Gamma , \quad \sigma _n' \le f \le \tau _n'. \end{aligned}$$

If $n\ge N$, then $0\le \sigma _n' - \sigma _N' \le f - \sigma _N \le \tau _N - \sigma _N$, whence $\Vert \varphi (\sigma _n') - \varphi (\sigma _N')\Vert \le \Vert \varphi (\tau _N) - \varphi (\sigma _N) \Vert \le N^{-1}$. Thus, the sequence $(\varphi (\sigma _n'))_{n\in \mathbb {N}}$ converges in the sense of the norm. So does $(\varphi (\tau _n'))_{n\in \mathbb {N}}$. Their limits are the same element *a* of *E*, and, since $\sigma _n' \uparrow , \tau _m' \downarrow$, we see that $a= \sup _{n\in \mathbb {N}}\varphi (\sigma _n') = \inf _{m\in \mathbb {N}}\varphi (\tau _m')$. $\square$

### **3.14**

**In the rest of this section** $\varvec{\Omega }$ **is the collection** $\mathbf{F^X}$ **of all maps of a set** $\mathbf{X}$ **into a partially ordered vector space** $\mathbf{F}$.

### **3.15**

A function $g: X \rightarrow \mathbb {R}$ determines a multiplication operator $f \mapsto gf$ in $\Omega$. We investigate the collection of all functions *g* for which

11 $$\begin{aligned} f\in \Gamma _V \quad \Longrightarrow \quad gf \in \Gamma _V, \end{aligned}$$

and, for given *f*, the behaviour of the map $g \mapsto \varphi _V(gf)$.

### **3.16**

For an algebra of subsets of *X*, $\mathcal {A}\subset \mathcal {P}(X)$ we write $[ \mathcal {A}]$ for the Riesz space of all $\mathcal {A}$-step functions, i.e., functions of the form $\sum _{i=1}^n \lambda _i \mathbbm {1}_{A_i}$ for $n\in \mathbb {N}$, $\lambda _i\in \mathbb {R}$, $A_i \in \mathcal {A}$ for $i\in \{1,\ldots ,n\}$. Define the collection of functions $[\mathcal {A}]^o$ by

12 $$\begin{aligned}{}[\mathcal {A}]^o =&\{ f\in \mathbb {R}^X: \text{ there } \text{ are } (s_n)_{n\in \mathbb {N}} \text{ in } [\mathcal {A}] \text{ and } (j_n)_{n\in \mathbb {N}} \text{ in } [\mathcal {A}]^{+} \nonumber \\&\text{ for } \text{ which } |f-s_n|\le j_n \text{ and } j_n \downarrow 0 \text{ pointwise }\}. \end{aligned}$$

(This $[\mathcal {A}]^o$ is the vertical extension of $[\mathcal {A}]$ obtained by, in Definition 3.3, choosing $E=\mathbb {R}^X,\Omega = \mathbb {R}^X, \Gamma = [\mathcal {A}], \ \varphi (f)=f \quad (f\in \Gamma )$.) Note that $[\mathcal {A}]$ and $[\mathcal {A}]^o$ are Riesz spaces, and uniform limits of elements of $[\mathcal {A}]$ are in $[\mathcal {A}]^o$. (Actually, $[\mathcal {A}]^o$ is uniformly complete.) Furthermore, $[\mathcal {A}]^o$ contains every bounded function *f* with $\{x\in X : f(x) \le s\} \in \mathcal {A}$ for all $s\in \mathbb {R}$. In case $\mathcal {A}$ is a $\sigma$-algebra, $[\mathcal {A}]^o$ is precisely the collection of all bounded $\mathcal {A}$-measurable functions.

### **Lemma 3.17**

Let $\mathcal {A}\subset \mathcal {P}(X)$ be an algebra of subsets of a set *X*. Suppose that $(g_n)_{n\in \mathbb {N}}$ is a sequence in $[\mathcal {A}]^o$ for which $g_n \downarrow 0$ pointwise. Then there exists a sequence $(j_n)_{n\in \mathbb {N}}$ in $[\mathcal {A}]$ with $j_n \ge g_n$ and $j_n \downarrow 0$ pointwise.

### *Proof*

For all $n\in \mathbb {N}$ there exists a sequence $(s_{nk})_{k\in \mathbb {N}}$ in $[\mathcal {A}]$ with $s_{nk} \ge g_n$ for all $k\in \mathbb {N}$ and $s_{nk} \downarrow _k g_n$ pointwise. Since $(g_n)_{n\in \mathbb {N}}$ is a decreasing sequence, we have $s_{mk} \ge g_n$ for all $m\le n$ and all $k\in \mathbb {N}$. Hence $j_n:=\inf _{m,k\le n } s_{mk}$ is an element in $[\mathcal {A}]$ with $j_n \ge g_n$. Clearly $j_n \downarrow$ and $\inf _{n\in \mathbb {N}}j_n = \inf _{n\in \mathbb {N}}\inf _{m,k\le n} s_{mk} = \inf _{n\in \mathbb {N}}\inf _{k\in \mathbb {N}}s_{nk} = \inf _{n\in \mathbb {N}}g_n =0$. $\square$

The following lemma is a consequence of Lemma 3.9.

### **Lemma 3.18**

Define the algebra

13 $$\begin{aligned} \mathcal {A}= \{ A \subset X: f\mathbbm {1}_A \in \Gamma \text{ for } f\in \Gamma \}. \end{aligned}$$

If $\varphi (\Gamma )$ is mediated in *E*, then

14 $$\begin{aligned} f\mathbbm {1}_A \in \Gamma _V \quad (f\in \Gamma _V, A\in \mathcal {A}). \end{aligned}$$

### **Definition 3.19**

*E* is called *Archimedean* 3 (see Peressini [8]) if for all $a,b\in E$ the following holds: if $na \le b$ for all $n\in \mathbb {N}$, then $a\le 0$.

### **Definition 3.20**

A sequence $(a_n)_{n\in \mathbb {N}}$ in *E* is called *order convergent* to an element $a\in E$ if there exists a sequence $(h_n)_{n\in \mathbb {N}}$ in $E^{+}$ with $h_n \downarrow 0$ and $- h_n \le a-a_n\le h_n$.

Notation: $$a_n \xrightarrow {o} a$$.

### **Theorem 3.21**

Let $\mathcal {A}$ be as in (13). Suppose that *E* is Archimedean, $\Gamma$ is directed and $\varphi (\Gamma )$ is mediated in *E*. Furthermore assume $\varphi$ has the following continuity property.

15 $$\begin{aligned}&\text{ If } A_1,A_2,\ldots \text{ in } ~\mathcal {A}~\text{ are } \text{ such } \text{ that } A_1 \supset A_2 \supset \cdots ~ \text{ and } ~\bigcap _{n\in \mathbb {N}} A_n = \emptyset , \nonumber \\&\text{ then }~ \varphi (f\mathbbm {1}_{A_n}) \downarrow 0 ~\text{ for } \text{ all }~ f\in \Gamma ^{+}. \end{aligned}$$

- $gf\in \Gamma _V$ for all $g\in [\mathcal {A}]^o$ and all $f\in \Gamma _V$.
- Let $g\in [\mathcal {A}]^o$ and let $(g_n)_{n\in \mathbb {N}}$ be a sequence in $[\mathcal {A}]^o$ for which there is a sequence $(j_n)_{n\in \mathbb {N}}$ in $[\mathcal {A}]^{o+}$ with $-j_n \le g_n -g \le j_n$ and $j_n \downarrow 0$ pointwise. Then

16 $$\begin{aligned} \varphi _V (g_n f) \xrightarrow {o} \varphi _V(gf) \quad (f\in \Gamma _V). \end{aligned}$$

(Order convergence in the sense of *E*.)

### *Proof*

We first prove the following:

($\star$) Let $f\in \Gamma _V^{+}$. Let $(g_n)_{n\in \mathbb {N}}$ be a sequence in $[\mathcal {A}]^o$ for which $g_n f \in \Gamma _V$ for all $n\in \mathbb {N}$ and $g_n \downarrow 0$ pointwise. Then

17 $$\begin{aligned} \varphi _V (g_n f) \downarrow 0. \end{aligned}$$

Let $\sigma \in \Gamma ^{+}$, $\sigma \ge f$. It follows from Lemma 3.17 that we may assume $g_n \in [\mathcal {A}]$ for all $n\in \mathbb {N}$. For all $n\in \mathbb {N}$ we have $0 \le \varphi _V(g_n f) \le \varphi _V( g_n \sigma )$, so we are done if $\varphi _V(g_n \sigma ) \downarrow 0$.

Let $h\in E$, $h\le \varphi _V(g_n \sigma )$ for all $n\in \mathbb {N}$; we prove $h\le 0$.

Take $\varepsilon >0$. For each $n\in \mathbb {N}$, set $A_n = \{x\in X: g_n(x) \ge \varepsilon \}$. Then $A_n\in \mathcal {A}$ for $n\in \mathbb {N}$ and $A_1 \supset A_2 \supset \cdots$ and $\bigcap _{n\in \mathbb {N}} A_n = \emptyset$. Putting $M= \Vert g_1\Vert _\infty$ we see that

18 $$\begin{aligned} g_n \le \varepsilon \mathbbm {1}_X + M \mathbbm {1}_{A_n} \quad (n\in \mathbb {N}), \end{aligned}$$

whence

19 $$\begin{aligned} h\le \varphi _V(g_n \sigma ) \le \varepsilon \varphi ( \sigma ) + M \varphi (\mathbbm {1}_{A_n} \sigma ) \quad (n\in \mathbb {N}). \end{aligned}$$

By the continuity property of $\varphi$, $h\le \varepsilon \varphi (\sigma )$. As this is true for each $\varepsilon >0$ and *E* is Archimedean, we obtain $h\le 0$.

(a) Since $\Gamma _V$ is directed (see 3.11) it is sufficient to consider $f\in \Gamma _V^{+}$. Let $g\in [\mathcal {A}]^o$. There are sequences of step functions $(h_n)_{n\in \mathbb {N}}$ and $(j_n)_{n\in \mathbb {N}}$ for which $h_n \uparrow g$, $j_n \downarrow g$ and thus $j_n - h_n \downarrow 0$. By Lemma 3.18 $h_n f, j_n f \in \Gamma _V$ for all $n\in \mathbb {N}$. Then $h_n f \le g f \le j_n f$ for $n\in \mathbb {N}$ and $\inf _{n\in \mathbb {N}} \varphi _V((j_n - h_n)f)=0$ by ($\star$). By Lemmas 3.7 and 3.5(c) we obtain that $gf\in \Gamma _V$.

(b) It is sufficient to consider $f\in \Gamma _V^{+}$. By (a) we may also assume $g=0$. But then (b) follows from ($\star$). $\square$

### *Remark 3.22*

Consider the situation in Theorem 3.21. Suppose $\mathcal {B}\subset \mathcal {A}$ is a $\sigma$-algebra. Then all bounded $\mathcal {B}$-measurable functions lie in $[\mathcal {A}]^o$. If $(g_n)_{n\in \mathbb {N}}$ is a bounded sequence of bounded $\mathcal {B}$-measurable functions that converges pointwise to a function *g*, then the condition of Theorem 3.21(b) is satisfied.

### *Remark 3.23*

In the next section we will consider a situation similar to the one of Theorem 3.21, in which $\mathcal {A}$ is replaced by a subset $\mathcal {I}$ that is closed under taking finite intersections. We will also adapt the continuity property on $\varphi$ (see 4.3).

## The lateral extension

The construction described in Definition 3.3 is reminiscent of the Riemann integral and, indeed, the Riemann integral is a special case (see Example 3.4).

In the present section we consider a type of extension, analogous to the *improper* Riemann integral. One usually defines the improper integral of a function *f* on $[0,\infty )$ to be

20 $$\begin{aligned} \lim _{s\rightarrow \infty } \int _0^s f(x) {{\mathrm{\, \mathrm {d}}}} x, \end{aligned}$$

approximating the domain, not the values of *f*.

For our purposes a more convenient description of the same integral would be

21 $$\begin{aligned} \sum _{n=1}^\infty \int _{a_n}^{a_{n+1}} f(x) {{\mathrm{\, \mathrm {d}}}} x, \end{aligned}$$

where $0=a_1<a_2<\cdots$ and $a_n \rightarrow \infty$. Here the domain is split up into manageable pieces.

Splitting up the domain is the basic idea we develop in this section. (This may explain our use of the terms “vertical” and “lateral”.)

**Throughout this section**, $\mathbf{X}$ **is a set**, $\mathbf{E}$ **and** $\mathbf{F}$ **are partially ordered vector spaces**, $\varvec{\Gamma }$ **is a directed** 4 **linear subspace of** $\mathbf{F^X}$, **and** $\varvec{\varphi }$ **is a linear order preserving map** $\varvec{\Gamma \rightarrow E}$. (**With** ${\varvec{\Omega }} = {\mathbf {F}}^{\mathbf {X}}$, **all considerations of Sect**.  3 **are applicable**.)

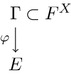



**Furthermore**, $\mathcal {I}$ **is a collection of subsets of** *X*, **closed under taking finite intersections. See Definitions** 4.1 **and** 4.2 **for two more assumptions.**

As a shorthand notation, if $(a_n)_{n\in \mathbb {N}}$ is a sequence in $E^{+}$ and $\{ \sum _{n=1}^N a_n : N\in \mathbb {N}\}$ has a supremum, we denote this supremum by

22 $$\begin{aligned} \sum _n a_n. \end{aligned}$$

### **Definition 4.1**

A disjoint sequence $(A_n)_{n\in \mathbb {N}}$ of elements in $\mathcal {I}$ whose union is *X* is called a *partition* If $(A_n)_{n\in \mathbb {N}}$ and $(B_n)_{n\in \mathbb {N}}$ are partitions and for all $n\in \mathbb {N}$ there exists an $m\in \mathbb {N}$ for which $B_n \subset A_m$, then $(B_n)_{n\in \mathbb {N}}$ is called a *refinement* of $(A_n)_{n\in \mathbb {N}}$. Note that if $(A_n)_{n\in \mathbb {N}}$ and $(B_n)_{n\in \mathbb {N}}$ are partitions then there exists a refinement of both $(A_n)_{n\in \mathbb {N}}$ and $(B_n)_{n\in \mathbb {N}}$ (e.g., a partition that consists of all sets of the form $A_n \cap B_m$ with ${n,m}\in {\mathbb {N}}$). **We assume that there exists at least one partition**.

### **Definition 4.2**

We call a linear subspace $\Delta$ of $F^X$ *stable* (under $\mathcal {I}$) if

23 $$\begin{aligned} f\mathbbm {1}_A\in \Delta \quad (f\in \Delta , A\in \mathcal {I}). \end{aligned}$$

If $\Delta$ is a stable space, then a linear and order preserving map $\omega : \Delta \rightarrow E$ is said to be *laterally extendable* if for all partitions $(A_n)_{n\in \mathbb {N}}$

24 $$\begin{aligned} \omega (f) = \sum _n \omega (f\mathbbm {1}_{A_n}) \quad \left( \text{ see } (22) \right) \quad (f\in \Delta ^{+}). \end{aligned}$$

**We assume** $\varvec{\Gamma }$ **is stable and** $\varvec{\varphi }$ **is laterally extendable**.

### **4.3**

In the situation of Theorem 3.21 we can choose $\mathcal {I}=\mathcal {A}$; then (15) is precisely the lateral extendability of $\varphi$.

### *Example 4.4*

For any partially ordered vector space *F* and a linear subspace $E\subset F$, the following choices lead to a system fulfilling all of our assumptions: $X= \mathbb {N}$, $\mathcal {I}= \mathcal {P}(\mathbb {N})$, $\Gamma = c_{00}[E]$ (see Sect. 2), $\varphi (f) = \sum _{n\in \mathbb {N}} f(n)$ for $f\in \Gamma$.

### **Definition 4.5**

Let $\Delta$ be a stable subspace of $F^X$ and let $\omega : \Delta \rightarrow E$ be a laterally extendable linear order preserving map. Let $(A_n)_{n\in \mathbb {N}}$ be a partition, and $f: X \rightarrow F$. We call $(A_n)_{n\in \mathbb {N}}$ a *partition for* *f* (occasionally $\Delta$ *-partition for* *f*) if

25 $$\begin{aligned} f \mathbbm {1}_{A_n} \in \Delta \quad (n\in \mathbb {N}). \end{aligned}$$

A function $f:X \rightarrow F$ is said to be a *partially in* $\Delta$ if there exists a partition for *f*. For $f: X \rightarrow F^{+}$, $(A_n)_{n\in \mathbb {N}}$ is called a $\omega$ *-partition for* *f* if it is a partition for *f* and if

26 $$\begin{aligned} \sum _{n} \omega (f \mathbbm {1}_{A_n}) \text{ exists }. \end{aligned}$$

A function $f: X \rightarrow F^{+}$ that is partially in $\Delta$ is called *laterally* $\omega$ *-integrable* if there exists a $\omega$-partition for *f*.

### *Example 4.6*

Consider the situation of Example 4.4. A function $x: \mathbb {N}\rightarrow F$ is partially in $\Gamma$ if and only if $x_n \in E$ for every $n\in \mathbb {N}$. If $x \ge 0$, then *x* is laterally integrable if $x_n \in E$ for every $n\in \mathbb {N}$ and $\sum _n x_n$ exists in *E*.

### **4.7**

Naturally, we wish to use (26) to define an integral for *f*. For that we have to show the supremum to be independent of the choice of the partition $(A_n)_{n\in \mathbb {N}}$.

### **Lemma 4.8**

- Let $f: X \rightarrow F$ and let $(A_n)_{n\in \mathbb {N}}$ be a partition for *f*. If $(B_n)_{n\in \mathbb {N}}$ is a partition that is a refinement of $(A_n)_{n\in \mathbb {N}}$, then $(B_n)_{n\in \mathbb {N}}$ is a partition for *f*.
- Let $f: X \rightarrow F^{+}$ and let $(A_n)_{n\in \mathbb {N}}$ and $(B_m)_{m\in \mathbb {N}}$ be partitions for *f*. Then the sets

27 $$\begin{aligned} \left\{ \sum _{n=1}^N \varphi (f\mathbbm {1}_{A_n}) : N\in \mathbb {N}\right\} \text{ and } \left\{ \sum _{m=1}^M \varphi (f\mathbbm {1}_{B_m}) : M\in \mathbb {N}\right\} \end{aligned}$$

have the same upper bounds in *E*.

### *Proof*

We leave the proof of (a) to the reader. Let *u* be an upper bound for the set $\{ \sum _{n=1}^N \varphi (f\mathbbm {1}_{A_n}) : N\in \mathbb {N}\}$; it suffices to prove that *u* is an upper bound for $\{\sum _{m=1}^M \varphi (f\mathbbm {1}_{B_m}) : M\in \mathbb {N}\}$. Take $M\in \mathbb {N}$; we are done if $u \ge \sum _{m=1}^M \varphi (f\mathbbm {1}_{B_m})$, i.e., if $u \ge \varphi (f\mathbbm {1}_B)$ where $B= B_1 \cup \cdots \cup B_M$. But $f\mathbbm {1}_B\in \Gamma$ so $\varphi (f\mathbbm {1}_B) = \sum _n \varphi (f\mathbbm {1}_B \mathbbm {1}_{A_n}) = \sup _{N\in \mathbb {N}}\sum _{n=1}^N \varphi (f\mathbbm {1}_B \mathbbm {1}_{A_n})$, whereas, for each $N\in \mathbb {N}$

28 $$\begin{aligned} \sum _{n=1}^N \varphi (f\mathbbm {1}_B \mathbbm {1}_{A_n}) \le \sum _{n=1}^N \varphi (f\mathbbm {1}_{A_n}) \le u. \end{aligned}$$

$$\square $$

### **Theorem 4.9**

Let $f:X \rightarrow F^{+}$ be laterally $\varphi$-integrable. Then every partition for *f* is a $\varphi$-partition for *f*. There exists an $a\in E^{+}$ such that for every partition $(A_n)_{n\in \mathbb {N}}$ for *f*,

29 $$\begin{aligned} a= \sum _n \varphi (f \mathbbm {1}_{A_n}). \end{aligned}$$

If $$f\in \Gamma ^{+}$$, then $$a= \varphi (f)$$.

### *Proof*

This is a consequence of Lemma 4.8(b). $\square$

### **Definition 4.10**

For a laterally $\varphi$-integrable $f: X \rightarrow F^{+}$ we call the element $a\in E^{+}$ for which (29) holds its $\varphi _L$ *-integral* and denote it by $\varphi _L(f)$. For the moment, denote by $(\Gamma ^{+})_L$ the set of all laterally $\varphi$-integrable functions $f: X \rightarrow F^{+}$. We proceed to extend $\varphi _L$ to a linear function defined on the linear hull of $(\Gamma ^{+})_L$, see Definition 4.14.

### **4.11**

The assumptions that $\Gamma$ is stable and $\varphi$ is laterally extendable are crucial for the fact that the $\varphi _L$-integral of a laterally $\varphi$-integrable function is independent of the choice of a $\varphi$-partition (see Lemma 4.8(b)).

### **4.12**

We will use the following rules for a partially ordered vector space *E*:

30 $$\begin{aligned} a_n \uparrow a, b_n \uparrow b&\Longrightarrow a_n + b_n \uparrow a + b&(a_n, b_n,a,b \in E), \end{aligned}$$

31 $$\begin{aligned} a_n \uparrow , \ b_n \uparrow b, \ a_n + b_n \uparrow a + b&\Longrightarrow a_n \uparrow a&(a_n, b_n,a,b \in E). \end{aligned}$$

### **4.13**

(Extending $$\varphi _L)$$ Define $$\Gamma _L= \{ f_1-f_2 : f_1,f_2\in (\Gamma ^{+})_L\}$$.

*Step 1* Let $f,g\in (\Gamma ^{+})_L$. There exists an $(A_n)_{n\in \mathbb {N}}$ that is a $\varphi$-partition for *f* and for *g*. By defining $a_N= \sum _{n=1}^N \varphi (f\mathbbm {1}_{A_n})$ and $b_N=\sum _{n=1}^N \varphi (g\mathbbm {1}_{A_n})$ for $N\in \mathbb {N}$, by (30) we obtain $f+g\in (\Gamma ^{+})_L$ with $\varphi _L(f+g) = \varphi _L(f) + \varphi _L(g)$.

Consequently, $\Gamma _L$ is a vector space, containing $(\Gamma ^{+})_L$.

*Step 2* If $g_1,g_2,h_1,h_2\in (\Gamma ^{+})_L$ and $g_1 -g_2 = h_1 - h_2$, then $g_1 + h_2 = g_2 + h_1$ so that, by the above, $\varphi _L(g_1) - \varphi _L(h_1) = \varphi _L(g_2) - \varphi _L(h_2)$.

Hence, $\varphi _L$ extends to a linear function $\Gamma _L \rightarrow E$ (also denoted by $\varphi _L$).

*Step 3* Let $f,g \in (\Gamma ^{+})_L$ and $f\le g$. By defining $a_N$ and $b_N$ as in step 1 and $c_N=b_N - a_N$, by (31) we infer that $g-f\in (\Gamma ^{+})_L$.

Thus, if $f\in \Gamma _L$ and $f\ge 0$, then $f\in (\Gamma ^{+})_L$. Briefly: $(\Gamma ^{+})_L$ is $\Gamma _L^{+}$, the positive part of $\Gamma _L$.

### **Definition 4.14**

A function $f: X \rightarrow F$ is called *laterally* $\varphi$ *-integrable* if $f\in \Gamma _L$ (see 4.13), i.e., if there exist $f_1,f_2\in (\Gamma ^{+})_L$ for which $f= f_1 - f_2$. The $\varphi _L$-integral of such a function is defined by $\varphi _L(f) = \varphi _L(f_1) - \varphi _L(f_2)$.

$\varphi _L$ is a function $\Gamma _L \rightarrow E$ and is called the *lateral extension* of $\varphi$. The set of laterally $\varphi$-integrable functions, $\Gamma _L$, is called the *lateral extension* of $\Gamma$ under $\varphi$.

Note that, thanks to Step 3 of 4.13, this definition of “laterally $\varphi$-integrable” does not conflict with the one given in Definition 4.10.

### **4.15**

Like for the vertical extension, we have the following elementary observations:

- $\Gamma \subset \Gamma _L$ 5 and $\varphi _L(\tau ) = \varphi (\tau )$ for all $\tau \in \Gamma$.
- $\Gamma _L$ is a directed partially ordered vector space and $\varphi _L$ is a linear order preserving function on $\Gamma _L$.
- If $\Pi$ is a directed linear subspace of $F^X$ and $\Pi \subset \Gamma$, then $\Pi _L \subset \Gamma _L$.

$((\Gamma _L)_L$ is not so easy. See Theorem 4.18 and Example 4.19.)

In case *E* is a Banach lattice with $\sigma$-order continuous norm, for $\Gamma _L^{+}$ we have an analogue of Theorem 3.13.

### **Lemma 4.16**

Suppose *E* is a Banach lattice with $\sigma$-order continuous norm. Let $f: X \rightarrow F^{+}$. Then *f* lies in $\Gamma _L^{+}$ if and only if there exists a $\Gamma$-partition $(A_n)_{n\in \mathbb {N}}$ for *f* such that the sequence $(\varphi (f\mathbbm {1}_{A_n}))_{n\in \mathbb {N}}$ has a sum in the sense of the norm, in which case $\varphi _L(f)$ is this sum.

### *Proof*

The “only if” part follows by definition of $\Gamma _L$ and the $\sigma$-order continuity of the norm. For the “if” part; this follows from the fact that if $a_n \uparrow$ and $\Vert a_n - a\Vert \rightarrow 0$ for $a,a_1,a_2,\ldots \in E$, then $a_n \uparrow a$. $\square$

We will now investigate conditions under which $\varphi _L$ and $\varphi _V$ themselves are laterally extendable. (For that, their domains have to be able to play the role of $\Gamma$, so they have to be stable.) First a useful lemma:

### **Lemma 4.17**

Let $f\in \Gamma _L$. Then there exists a partition $(A_n)_{n\in \mathbb {N}}$ for *f* such that every refinement $(B_m)_{m\in \mathbb {N}}$ of it (is a partition for *f* and) has this property:

32 $$\begin{aligned} h\in E, \ h \ge \sum _{m=1}^M \varphi (f\mathbbm {1}_{B_m}) \text{ for } \text{ all } M\in \mathbb {N}\quad \Longrightarrow \quad h\ge \varphi _L(f). \end{aligned}$$

### *Proof*

Write $f= f_1 - f_2$ with $f_1, f_2 \in \Gamma _L^{+}$. Let $(A_n)_{n\in \mathbb {N}}$ be a partition for $f_1$ and $f_2$, and let $(B_m)_{m\in \mathbb {N}}$ be a refinement of $(A_n)_{n\in \mathbb {N}}$. Note that $(B_m)_{m\in \mathbb {N}}$ is a partition for $f_1$ and $f_2$. Let *h* be an upper bound for $\{ \sum _{m=1}^M \varphi (f\mathbbm {1}_{B_m}): M\in \mathbb {N}\}$ in *E*. For all $M\in \mathbb {N}$,

33 $$\begin{aligned} h+ \sum _{m=1}^M \varphi (f_2 \mathbbm {1}_{B_m}) \ge \sum _{m=1}^M \varphi (f\mathbbm {1}_{B_m}) + \sum _{m=1}^M \varphi (f_2 \mathbbm {1}_{B_m}) = \sum _{m=1}^M \varphi (f_1 \mathbbm {1}_{B_m}). \end{aligned}$$

Taking the supremum over *M* yields $h + \varphi _L(f_2) \ge \varphi _L(f_1)$, i.e., $h\ge \varphi _L(f)$. $\square$

### **Theorem 4.18**

- Suppose $\Gamma _L$ is stable. Then $\varphi _L$ is laterally extendable, i.e.,
  34 $$\begin{aligned} \varphi _L(f) = \sum _n \varphi _L (f \mathbbm {1}_{A_n}) \end{aligned}$$
  for every $f\in \Gamma _L^{+}$ and every $\varphi _L$-partition $(A_n)_{n\in \mathbb {N}}$ for *f*. Therefore $\left( \Gamma _L\right) _L= \Gamma _L$ and $(\varphi _L)_L = \varphi _L$.
- Suppose $\Gamma _V$ is stable. Then $\varphi _V$ is laterally extendable. (For $(\Gamma _V)_L$ see Sect. 5.)

### *Proof*

- Let $f\in \Gamma _L^{+}$ and let $(B_n)_{n\in \mathbb {N}}$ be a $\varphi _L$-partition for *f*. Let $(A_n)_{n\in \mathbb {N}}$ be the partition for *f* as in Lemma 4.17. Then form a common refinement of $(B_n)_{n\in \mathbb {N}}$ and $(A_n)_{n\in \mathbb {N}}$ and apply Lemma 4.17.
- Let $f\in \Gamma _V^{+}$ and let $(A_n)_{n\in \mathbb {N}}$ be a partition. Let $h\in E, h \ge \sum _{n=1}^N \varphi _V(f \mathbbm {1}_{A_n})$ for every $N\in \mathbb {N}$. We wish to prove $h \ge \varphi _V(f)$, which will be the case if $h \ge \varphi (\sigma )$ for every $\sigma \in \Gamma$ with $\sigma \le f$. For that apply Lemma 4.17 to $\sigma$.

$$\square $$

The following shows that $\Gamma _L$ may not be stable, in which case there is no $(\Gamma _L)_L$. (However, see Theorem 4.25(a).)

### *Example 4.19*

Consider the situation in Example 4.4 and assume there is an $a: \mathbb {N}\rightarrow E^{+}$ such that $\sum \nolimits _n a_n$ exists in *F* and $\sum \nolimits _n a_{2n}$ does not (e.g. $E=F=c$ and $a_n=e_n = \mathbbm {1}_{\{n\}}$). By Example 4.6 *a* lies in $\Gamma _L$ but $b= (0,a_2,0,a_4,\ldots )$ does not; but $b = a \mathbbm {1}_{\{2,4,6,\ldots \}}$ and $\{2,4,6,\ldots \}\in \mathcal {I}$. (Actually, the existence of such an $a: \mathbb {N}\rightarrow E^{+}$ is equivalent to *E* not being “splitting” in *F*; see Definition 4.21 and (36).)

### *Remark 4.20*

$\Gamma _V$ may not be stable either. With $E=c$, $F= \ell ^\infty$, $X= \{1,2\}$, $\Gamma = c\times c$ and $\varphi (f,g)=f +g$ (as in Example 3.8), the space $\Gamma _V$ is not stable for $\mathcal {I}= \mathcal {P}(X)$.

### **Definition 4.21**

Let *D* be a linear subspace of *E*. *D* is called *splitting* in *E* if the following is true:

35 $$\begin{aligned}&\text{ If } (a_n)_{n\in \mathbb {N}}\,\text{ and }\,(b_n)_{n\in \mathbb {N}}\,\text{ are } \text{ sequences } \text{ in }\,D\,\text{ with }\,0\le a_n \le b_n\,\text{ for }\,n\in \mathbb {N}\nonumber \\&\text{ and }\,\sum \nolimits _n b_n\,\text{ exists } \text{ in }\,E,\,\text{ then } \text{ so } \text{ does }\,\sum \nolimits _n a_n. \end{aligned}$$

It is not difficult to see that *D* is splitting in *E* if and only if

36 $$\begin{aligned}&\text{ If } (a_n)_{n\in \mathbb {N}}\,\text{ is } \text{ a } \text{ sequence } \text{ in }\,D^{+}\,\text{ and }\,\sum \nolimits _n a_n\,\text{ exists } \text{ in }\,E, \nonumber \\&\text{ then } \text{ so } \text{ does }\,\sum \nolimits _n \mathbbm {1}_A(n) a_n\,\text{ for } \text{ all }\,A\subset \mathbb {N}. \end{aligned}$$

If *D* is splitting in *E*, then so is every linear subspace of *D*. If *E* is $\sigma$-Dedekind complete, then *E* is also splitting. More generally, *D* is splitting in *E* if every bounded increasing sequence in *D* has a supremum in *E*. Also, $\mathbb {R}^2$ with the lexicographical ordering is splitting.

In Theorem 4.25 we will see what is the use of this concept. First, we have a look at the connection between “splitting” and “mediated”.

### **Lemma 4.22**

Suppose *D* is a linear subspace of *E*. Consider the condition:

37 $$\begin{aligned}&\text{ For } \text{ all } \text{ sequences } (a_n)_{n\in \mathbb {N}}, (b_n)_{n\in \mathbb {N}} \text{ in } D: \nonumber \\&a_n \downarrow , b_n \uparrow , \ \inf _{n\in \mathbb {N}}a_n- b_n=0 \ \ \Longrightarrow \ \ \inf _{n\in \mathbb {N}}a_n = \sup _{n\in \mathbb {N}}b_n. \end{aligned}$$

(The infima and suprema in (37) are to be taken in *E*.) If *D* is either splitting or mediated in *E*, then (37) holds. Conversely, (37) implies that *D* is splitting if $D=E$, whereas (37) implies that *D* is mediated in *E* if *E* is a Riesz space and *D* is a Riesz subspace of *E*.

### *Proof*

It will be clear that mediatedness implies (37) and vice versa if *E* is a Riesz space and *D* a Riesz subspace of *E*.

If *D* is splitting in *E* and $a_n\downarrow , b_n \uparrow$ and $\inf a_n - b_n =0$, then $\sum _n b_{n+1}- b_n + a_{n} - a_{n+1} = a_1 - b_1$. Hence (37) holds.

Suppose $D=E$ and (37) holds. Let $(a_n)_{n\in \mathbb {N}}$ and $(b_n)_{n\in \mathbb {N}}$ be sequences in *D* with $0\le a_n \le b_n$ for $n\in \mathbb {N}$ such that $\sum _n b_n$ exists. Let $z = \sum _n b_n$, $A_n = \sum _{i=1}^n a_i$, $C_n = \sum _{i=1}^n b_i - a_i$ for $n\in \mathbb {N}$. Then $A_n \uparrow , C_n \uparrow$ and $z- C_n - A_n \downarrow 0$ (note that $z- C_n \in D$). Hence $\sup _{n\in \mathbb {N}}A_n = \sum _n a_n$ exists. $\square$

### **4.23**

- If *E* is a Riesz space, then every splitting Riesz subspace is mediated in *E*.
- If *E* is mediated, then it is splitting. The converse is also true if *E* is a Riesz space.
- $c_{00}$ is mediated in *c*, not splitting in *c* (with $D=E=c$ also (37) is not satisfied).
- If *D* is the space of all polynomial functions on [0, 1] with degree at most 2 and $$E=C[0,1]$$, then *D* is splitting in *E*, but not mediated in *E*. (Actually, *D* is splitting, but not mediated.) *D* is splitting (and satisfies (37) with $$E=D)$$: if $$u_n\in E^{+}$$, $$u_n \uparrow $$ and $$u_n \le \mathbbm {1}$$, then $$|u_n(x)- u_n(y)|\le 4|x-y|$$ as can be concluded from the postscript in Example 5.15. Therefore the pointwise supremum is continuous. It is even in *D* since $$u_n(x) = a_n x^2 + b_n x + c_n$$, where $$a_n, b_n,c_n$$ are linear combinations of $$u_n(0),u_n(\frac{1}{2}), u_n(1)$$ (see also the postscript in Example 5.15). *D* is not mediated: for example one can find countable $$A,B \subset E$$ for which $$\mathbbm {1}_{[\frac{1}{2},1]}$$ is pointwise the infimum of *A* and $$\mathbbm {1}_{(\frac{1}{2},1]}$$ is pointwise the supremum of *B*, then $$\inf A- B=0$$, but there is no $$h\in E$$ with $$B\le h \le A$$.

### **Theorem 4.24**

Let *E* be a Banach lattice with $\sigma$-order continuous norm. Then *E* is both mediated and splitting.

### *Proof*

Suppose $a_n,b_n\in E$ with $0 \le a_n \le b_n$ for $n\in \mathbb {N}$. Suppose that $\{\sum _{n=1}^N b_n: N\in \mathbb {N}\}$ has a supremum *s* in *E*. We prove that $\{\sum _{n=1}^N a_n: N\in \mathbb {N}\}$ has a supremum in *E*. Since the norm is $\sigma$-order continuous, we have $\Vert s- \sum _{n=1}^N b_n\Vert \rightarrow 0$. In particular we get that for all $\varepsilon >0$ there exists an $N\in \mathbb {N}$ such that for all $n,m\ge N$ with $m>n$ we have $\Vert \sum _{i=n}^m b_i\Vert <\varepsilon$ and thus $\Vert \sum _{i=n}^m a_i\Vert <\varepsilon$. From this we infer that $(\sum _{n=1}^N a_n)_{N\in \mathbb {N}}$ converges in norm. Therefore it has a supremum in *E*. Thus *E* is splitting. By Lemma 4.22 *E* is mediated. $\square$

### **Theorem 4.25**

- $\varphi (\Gamma )$ splitting in *E* $\Longrightarrow$ $\Gamma _L$ is stable and $\varphi _L$ is laterally extendable.
- $\varphi (\Gamma )$ mediated in *E* $\Longrightarrow$ $\Gamma _V$ is stable and $\varphi _V$ is laterally extendable.
- $\varphi (\Gamma )$ splitting in *E* and $\varphi _L(\Gamma _L)$ mediated in *E* $\Longrightarrow$ $(\Gamma _{L})_V$ is stable and $(\varphi _{L})_V$ is laterally extendable.

### *Proof*

- Let $f\in \Gamma _L$, $B\in \mathcal {I}$; we prove $f\mathbbm {1}_B \in \Gamma _L$. (This is sufficient by Theorem 4.18(a).) Without loss of generality, assume $f\ge 0$. Choose a $\varphi$-partition $(A_n)_{n\in \mathbb {N}}$ for *f*. Now apply (35) to
  38 $$\begin{aligned} a_n := \varphi ( f \mathbbm {1}_{A_n \cap B}), \quad b_n:= \varphi (f\mathbbm {1}_{A_n}) \quad (n\in \mathbb {N}). \end{aligned}$$
- follows from Lemma 3.18 and Theorem 4.18(b).
- By (a) $\Gamma _L$ is stable and $\varphi _L$ is laterally extendable. Hence we can apply (b) to $\Gamma _L$ and $\varphi _L$ (instead of $\Gamma$ and $\varphi$) and obtain (c).

$$\square $$

### **4.26**

To some extent, the assumption of Theorem 4.25(a) is minimal.

Indeed, in the situation of Example 4.4, we see that $\Gamma _L$ is stable if and only if *E* (which is $\varphi (\Gamma ))$ is splitting in *F* (see (36)).

In Theorem 4.25(c) we assumed that $\varphi _L(\Gamma _L)$ (and thus also $\varphi (\Gamma )$) was mediated in *E*. It may happen that $\varphi (\Gamma )$ is mediated in *E*, but $\varphi _L(\Gamma _L)$ is not, as Example 4.27 illustrates. However, splitting is preserved under the lateral extension and mediation is preserved under the vertical extension, see Theorem 4.28.

### *Example 4.27*

Let $X=\mathbb {N}$, $\mathcal {I}= \mathcal {P}(\mathbb {N})$, $E=F=c$. Let $\Gamma = c_{00}[c_{00}]$ (see Sect. 2) and $\varphi : \Gamma \rightarrow E$ be given by $\varphi (f) = \sum _{n\in \mathbb {N}} f(n)$. Then $\varphi (\Gamma )= c_{00}$, which is mediated in *c*. A function $f: \mathbb {N}\rightarrow c$ is partially in $\Gamma$ if and only if $f(\mathbb {N}) \subset c_{00}$. For $x\in c^{+}$ the function given by $f(n) = x(n) \mathbbm {1}_{\{n\}}$ for $n\in \mathbb {N}$ lies in $\Gamma _L$, and $\varphi _L(f) =x$. It follows that $\varphi _L(\Gamma _L)$ is *c*, which is not mediated in *c*.

### **Theorem 4.28**

- If $\varphi (\Gamma )$ is splitting in *E*, then so is $\varphi _L(\Gamma _L)$.
- If $\varphi (\Gamma )$ is mediated in *E*, then so is $\varphi _V(\Gamma _V)$.

### *Proof*

- Suppose $a_n\in \varphi _L(\Gamma _L)^{+}$ for $n\in \mathbb {N}$ and $\sum _n a_n$ exists. Let $A\subset \mathbb {N}$. For all $n\in \mathbb {N}$ there exist $b_{n1},b_{n2},\ldots \in \varphi (\Gamma )^{+}$ with $a_n = \sum _m b_{nm}$. Hence $\sum _n a_n = \sum _{n,m} b_{nm}$ and so $\sum _{n,m} \mathbbm {1}_{A\times \mathbb {N}}(n,m) b_{nm} = \sum _n \mathbbm {1}_A(n) a_n$ exists in *E*.
- Suppose $A,B\subset \varphi _V(\Gamma _V)$ are countable sets with $\inf A-B=0$. For all $a\in A$ and $b\in B$ there exist countable sets $\Upsilon _a,\Lambda _b\subset \Gamma$ with $a= \inf \varphi (\Upsilon _a), b= \sup \varphi (\Lambda _b)$. Then $\inf \varphi ( \bigcup _{a\in A} \Upsilon _a - \bigcup _{b\in B} \Lambda _b)=0$ and thus $\inf A = \inf \varphi (\bigcup _{a\in A} \Upsilon _a )= \sup \varphi ( \bigcup _{b\in B} \Lambda _b)= \sup B$.

$$\square $$

### **4.29**

For a Riesz space *F* we will now investigate under which conditions the space $\Gamma _L$ is a Riesz subspace of $F^X$. The next example shows that even if *E* is a Riesz space and $\Gamma$ is a Riesz subspace of $F^X$, $\Gamma _L$ may not be one. However, see Theorem 4.32.

### *Example 4.30*

Let *a*, *b* be as in Example 4.19; this time put $d=(0,a_1+a_2,0,a_3+a_4,\ldots )$. Then $a,d\in \Gamma _L$ but $a\wedge d = b \notin \Gamma _L$.

Hence, in Example 4.4, if *F* is a Riesz space and *E* is not splitting in *F*, then $\Gamma _L$ is not a Riesz subspace of $F^X$. As we will see in Theorem 4.32, considering the situation of Example 4.4: $\Gamma _L$ is a Riesz subspace of $F^X$ if and only if *E* is splitting in *F*.

### **Lemma 4.31**

Let $f: X \rightarrow F$ be partially in $\Gamma$.

- If *f* is in $\Gamma _{LV}$, then $f \in \Gamma _L$.
- Suppose $\varphi (\Gamma )$ is splitting in *E*. If $g\le f \le h$ for certain $g,h\in \Gamma _L$, then $f\in \Gamma _L$.

### *Proof*

- By the definition of $\Gamma _{LV}$ there exists a $\rho \in \Gamma _L$ with $\rho \le f$. Then $f- \rho$ is partially in $\Gamma$, $f-\rho \in \Gamma _{LV}$, and we are done if $f- \rho \in \Gamma _L$. Hence we may assume $f \ge 0$. Let $(A_n)_{n\in \mathbb {N}}$ be a partition for *f*; we prove $\sum _n \varphi (f\mathbbm {1}_{A_n}) = \varphi _{LV}(f)$. It will be clear that $\sum _{n=1}^N \varphi (f \mathbbm {1}_{A_n}) \le \varphi _{LV} ( f)$ for $N\in \mathbb {N}$. For the reverse inequality let $h\in E$ be an upper bound for $\{ \sum _{n=1}^N \varphi (f \mathbbm {1}_{A_n}): N\in \mathbb {N}\}$. It suffices to show that *h* must be an upper bound for $\{ \varphi _L(\sigma ): \sigma \in \Gamma _L, \sigma \le f\}$. Take a $\sigma \in \Gamma _L$ with $\sigma \le f$. If $(B_n)_{n\in \mathbb {N}}$ is any refinement of $(A_n)_{n\in \mathbb {N}}$ that is a $\varphi$-partition for $\sigma$, then for all $M\in \mathbb {N}$ there exists an $N\in \mathbb {N}$ with $B_1 \cup \cdots \cup B_M \subset A_1 \cup \cdots \cup A_N$, so that $h \ge \sum _{n=1}^N \varphi (f \mathbbm {1}_{A_n}) \ge \sum _{m=1}^M \varphi (f \mathbbm {1}_{B_m})\ge \sum _{m=1}^M \varphi (\sigma \mathbbm {1}_{B_m})$. It follows from Lemma 4.17, applied to $\sigma$, that the partition $(B_m)_{m\in \mathbb {N}}$ can be chosen so that this implies $h \ge \varphi _L(\sigma )$.
- As $h-g \in \Gamma _L$ and $0 \le f-g \le h-g$, we may (and do) assume $g=0$. Let $(A_n)_{n\in \mathbb {N}}$ be a partition for *f* that is also a $\varphi$-partition for *h*. Now just apply (35) to
  39 $$\begin{aligned} a_n:= \varphi (f \mathbbm {1}_{A_n}), \quad b_n:= \varphi (h\mathbbm {1}_{A_n}) \quad (n\in \mathbb {N}). \end{aligned}$$

$$\square $$

As a consequence of Lemma 4.31:

### **Theorem 4.32**

Let *F* be a Riesz space and $\Gamma$ be a Riesz subspace of $F^X$. The functions $X\rightarrow F$ that are partially in $\Gamma$ form a Riesz space, $\Xi$. If $\varphi (\Gamma )$ is splitting in *E*, then $\Gamma _L$ is a Riesz ideal in $\Xi$, in particular, $\Gamma _L$ is a Riesz space.

In the classical integration theory and the Bochner integration theory one starts with considering a measure space $(X,\mathcal {A},\mu )$ and simple functions on *X* with values in $\mathbb {R}$ or in a Banach space. One defines an integral on these simple functions using the measure and extends this integral to a larger class of integrable functions. In 4.33 we will follow a similar procedure, replacing $\mathbb {R}$ or the Banach space with *E* and applying the lateral extension. In Sect. 8 we will treat such extensions in more detail.

### **4.33**

Suppose $(X,\mathcal {A},\mu )$ is a $\sigma$-finite complete measure space and suppose *E* is directed. Let $F=E$. For $\mathcal {I}$ we choose $\{A\in \mathcal {A}: \mu (A)<\infty \}$. The $\sigma$-finiteness of $\mu$ guarantees the existence of a partition (and vice versa).

We say that a function $f: X\rightarrow E$ is *simple* if there exist $N\in \mathbb {N}$, $a_1,\ldots ,a_N\in E$, $A_1,\ldots , A_N\in \mathcal {I}$ for which

40 $$\begin{aligned} f = \sum _{n=1}^N a_n \mathbbm {1}_{A_n}. \end{aligned}$$

The simple functions form a stable directed linear subspace *S* of $E^X$, which is a Riesz subspace of $E^X$ in case *E* is a Riesz space.

For a given *f* in *S* one can choose a representation (40) in which the sets $A_1,\ldots , A_N$ are pairwise disjoint; thanks to the $\sigma$-finiteness of $\mu$ one can choose them in such a way that they occur in a partition $(A_n)_{n\in \mathbb {N}}$.

This *S* is going to be our $\Gamma$. We define $\varphi : S\rightarrow E$ by

41 $$\begin{aligned} \varphi (f) = \sum _{n=1}^N \mu (A_n) a_n, \end{aligned}$$

where $f,N,A_n,a_n$ are as in (40). The $\sigma$-additivity of $\mu$ is (necessary and) sufficient to show that *S* is laterally extendable.

A function $f: X\rightarrow E$ is partially in *S* if and only if there exist a partition $(A_n)_{n\in \mathbb {N}}$ and a sequence $(a_n)_{n\in \mathbb {N}}$ in *E* for which

42 $$\begin{aligned} f = \sum _{n\in \mathbb {N}} a_n \mathbbm {1}_{A_n}. \end{aligned}$$

An *f* as in (42) with $f\ge 0$ that is partially in *S* is an element of $S_L$ if and only if $\sum _n \mu (A_n) a_n$ exists in *E*. (See Theorem 4.9.)

## Combining vertical and lateral extensions

**In this section** $\mathbf {E,F,X},\varvec{\mathcal {I},\Gamma ,\varphi }$ **are as in Sect**. 4.

As we have seen, the lateral extension differs from the vertical extension in the sense that the vertical extensions of $\Gamma$ and $\varphi$ can always be made, but for lateral extension we had to assume the space $\Gamma$ to be stable and $\varphi$ to be laterally extendable (see 4.11). In this section we investigate when one can make a lateral extension of another (say vertical) extension. Furthermore we will compare different extensions and combinations of extensions.

Instead of $(\Gamma _L)_V$ and $((\Gamma _L)_V)_L$ we write $\Gamma _{LV}$ and $\Gamma _{LVL}$; similarly $\varphi _{LV}= (\varphi _{L})_V$ etc.

### **5.1**

By Theorem 4.18 the following holds for a stable directed linear subspace $\Delta$ of $F^X$ and a laterally extendable order preserving linear map $\omega : \Delta \rightarrow E$: If $\Delta _L$ is stable, then $\omega _L$ is laterally extendable (and so $\Delta _{LL}$ exists). If $\Delta _V$ is stable, then $\omega _V$ is laterally extendable (and so $\Delta _{VL}$ exists). We will use these facts without explicit mention.

### **5.2**

The following statements follow from the definitions and theorems we have:

- $$\Gamma _V \subset \Gamma _{LV}$$ and $$\varphi _{LV}=\varphi _V$$ on $$\Gamma _V$$.
- $$\Gamma _L \subset \Gamma _{LV}$$ and $$\varphi _{LV}=\varphi _L$$ on $$\Gamma _L$$.
- $\varphi _V= \varphi _L$ on $\Gamma _L\cap \Gamma _V$. For (d), (e) and (f) let $\Gamma _V$ be stable.
- $$\Gamma _{LV} \subset \Gamma _{VLV}$$ and $$\varphi _{VLV}=\varphi _{LV}$$ on $$\Gamma _{LV}$$.
- $$\Gamma _{VL} \subset \Gamma _{VLV}$$ and $$\varphi _{VLV} = \varphi _{VL}$$ on $$\Gamma _{VL}$$.
- $$\varphi _{LV}= \varphi _{VL}$$ on $$\Gamma _{LV} \cap \Gamma _{VL}$$.

Observe that as a consequence of (a) and (b): if $f\in \Gamma _L$ and $g\in \Gamma _V$ and $f\le g$ (or $f\ge g)$, then $\varphi _L(f) \le \varphi _V(g)$ (or $\varphi _L(f) \ge \varphi _V(g))$. Moreover, as a consequence of (c) and (d); if $\Gamma _V$ is stable: if $f\in \Gamma _{LV}$ and $g\in \Gamma _{VLV}$ and $f\le g$ (or $f\ge g)$, then $\varphi _{LV}(f) \le \varphi _{VLV}(g)$ (or $\varphi _{LV}(f) \ge \varphi _{VLV}(g))$.

### **5.3**

Note that if $\Gamma$ is stable and $\varphi$ is laterally extendable, then we can extend $\Gamma$ to $\Gamma _V, \Gamma _L$ and $\Gamma _{LV}$. If, moreover, $\Gamma _V$ is stable, then we can also extend $\Gamma$ to $\Gamma _{VL}$ and $\Gamma _{VLV}$. However, “more stability” will not give us larger extensions than $\Gamma _{VLV}$. Indeed, if $\Gamma _{LV}$ is stable then $\Gamma _{LV} \subset \Gamma _{LVL}=\Gamma _{VL}$ (see Theorem 5.8). If moreover $\Gamma _{VLV}$ is stable, then even $\Gamma _{VLVL}= \Gamma _{VLV}=\Gamma _{VL}$.

### **Lemma 5.4**

- If $f\in \Gamma _{LV}^{+}$, then there exists a countable $\Lambda \subset \Gamma$ with $\Lambda \le f$ and $\varphi _{LV}(f) = \sup \varphi (\Lambda )$.
- If $\Gamma _V$ is stable and $f\in \Gamma _{VL}^{+}$, then there exists a countable $\Lambda \subset \Gamma$ with $\Lambda \le f$ and $\varphi _{VL}(f) = \sup \varphi (\Lambda )$.

### *Proof*

- There exist $\sigma _1,\sigma _2, \ldots$ in $\Gamma _L$ with $\sigma _n \le f$ for all $n\in \mathbb {N}$ and $\sup _{n\in \mathbb {N}}\varphi _{L}(\sigma _n) = \varphi _{LV}(f)$. Hence, we are done if for every $\sigma$ in $\Gamma _L$ with $\sigma \le f$ there is a countable set $\Lambda _\sigma \subset \{ \rho \in \Gamma : \rho \le f\}$ such that every upper bound for $\varphi (\Lambda _\sigma )$ majorizes $\varphi _L(\sigma )$. But that is not hard to prove. For such a $\sigma$, by Lemma 4.17 there exists a partition $(B_m)_{m\in \mathbb {N}}$ for which (32) holds. Now let $\Lambda _\sigma$ be $\{\sum _{m=1}^M \sigma \mathbbm {1}_{B_m}: M\in \mathbb {N}\}$.
- Suppose $\Gamma _V$ is stable. Let $(A_n)_{n\in \mathbb {N}}$ be a $\varphi _V$-partition for *f*. Then the set $\Lambda _f=\{ \sum _{n=1}^N f \mathbbm {1}_{A_n}: N\in \mathbb {N}\}$ is a countable subset of $\Gamma _V$ and $\sup \varphi _V(\Lambda _f) = \varphi _{VL}(f)$. Moreover, for every $N\in \mathbb {N}$ there is a countable set $\Lambda _N \subset \{ \sigma \in \Gamma : \sigma \le \sum _{n=1}^N f\mathbbm {1}_{A_n}\}$ for which $\sup \varphi (\Lambda _N) = \varphi _{V}( \sum _{n=1}^N f\mathbbm {1}_{A_n})$. Take $\Lambda = \bigcup _{N\in \mathbb {N}} \Lambda _N$.

$$\square $$

### **Theorem 5.5**

For (b), (c), (d) and (e) let $\Gamma _V$ be stable and *f* be partially in $\Gamma _V$.

- If $$f\in \Gamma _{LV}$$, then^6^
  $$\begin{aligned} f\in \Gamma _V\iff \text{ there } \text{ exist } \pi ,\rho \in \Gamma \text{ with } \pi \le f \le \rho . \end{aligned}$$
- If $$f\in \Gamma _{VL}$$, then
  $$\begin{aligned} f\in \Gamma _V\iff \text{ there } \text{ exist } \pi ,\rho \in \Gamma \text{ with } \pi \le f \le \rho \ ^{6}. \end{aligned}$$
- $$ f \in \Gamma _{LV} \iff f\in \Gamma _{VL} \text{ and } \text{ there } \text{ exist } \pi ,\rho \in \Gamma _L \text{ with } \pi \le f \le \rho . $$
- If $\varphi _V(\Gamma _V)$ is splitting in *E*, then
  $$\begin{aligned} f\in \Gamma _{VL} \iff \text{ there } \text{ exist } \pi ,\rho \in \Gamma _{VL} \text{ with } \pi \le f \le \rho . \end{aligned}$$
- If $\varphi _V(\Gamma _V)$ is splitting in *E*, then
  $$\begin{aligned} f\in \Gamma _{VL}\cap \Gamma _{LV} \iff \text{ there } \text{ exist } \pi ,\rho \in \Gamma _{L} \text{ with } \pi \le f \le \rho . \end{aligned}$$

### *Proof*

The proofs of (a) and (b) are similar to the proof of (c) and therefore omitted.

(c) $\Leftarrow$: By Lemma 5.4 (b) there exist countable sets $\Lambda , \Upsilon \subset \Gamma$ with $\Lambda \le f- \pi$ and $\Upsilon \le \rho - f$ for which $\sup \varphi (\Lambda ) = \varphi _{VL}(f-\pi )$ and $\sup \varphi (\Upsilon ) = \varphi _{VL}(\rho - f)$. Then $\Lambda + \pi$ and $\rho - \Upsilon$ are countable subsets of $\Gamma _L$ with $\Lambda + \pi \le f \le \rho - \Upsilon$ and $\sup \varphi _L(\Lambda + \pi ) = \varphi _{VL}(f) = \inf \varphi _L (\rho - \Upsilon )$. Hence $f\in \Gamma _{LV}$.
$\Rightarrow$: Let $f\in \Gamma _{LV}$ and be partially in $\Gamma _V$. There exists a $\pi \in \Gamma _L$ for which $f- \pi \in \Gamma _{LV}^{+}$, hence we may assume $f\ge 0$. Let $(A_n)_{n\in \mathbb {N}}$ be a $\Gamma _V$-partition for *f*, i.e., $f\mathbbm {1}_{A_n}\in \Gamma _V$ and thus $\varphi _{LV}(f\mathbbm {1}_{A_n}) = \varphi _V(f\mathbbm {1}_{A_n})$ for all $n\in \mathbb {N}$ (see 5.2(a)). Then $\varphi _{LV}(f) \ge \sum _{n=1}^N \varphi _V(f\mathbbm {1}_{A_n})$ for all $N\in \mathbb {N}$. Let $h\in E$ be such that $h \ge \sum _{n=1}^N \varphi _V(f\mathbbm {1}_{A_n})$ for all $N\in \mathbb {N}$. From Lemma 4.17 we infer that $h\ge \varphi _L(\sigma )$ for every $\sigma \in \Gamma _L$ with $\sigma \le f$. We conclude that $\sum _n \varphi _V(f\mathbbm {1}_{A_n}) = \varphi _{LV}(f)$, i.e., $f\in \Gamma _{VL}$.
(d) $\Leftarrow$: We may assume $\pi =0$. Let $(A_n)_{n\in \mathbb {N}}$ be a $\varphi _V$-partition for $\rho$ with $f\mathbbm {1}_{A_n}\in \Gamma _V$ for all $n\in \mathbb {N}$. Then $0\le \varphi _V(f\mathbbm {1}_{A_n}) \le \varphi _V(\rho \mathbbm {1}_{A_n})$ for all $n\in \mathbb {N}$ and $\sum _{n} \varphi _V(\rho \mathbbm {1}_{A_n})$ exists in *E*. Hence, so does $\sum _{n} \varphi _V(f \mathbbm {1}_{A_n})$, i.e., $f\in \Gamma _{VL}$.
(e) is a consequence of (c) and (d).

$$\square $$

In the following example all functions in $\Gamma _{LV}$ are partially in $\Gamma _V$.

### *Example 5.6*

Consider $X=\mathbb {N}, \mathcal {I}=\mathcal {P}(\mathbb {N})$, $E=F$; let *D* be a linear subspace of *E* and let $D_V$ be the vertical extension of *D* with respect to the inclusion map $D\rightarrow E$. Let $\Gamma = c_{00}[D]$ and $\varphi : \Gamma \rightarrow E$ be $\varphi (f) = \sum _{n\in \mathbb {N}}f(n)$. Then $\Gamma _V = c_{00}[D_V]$. Let $f\in \Gamma _{LV}$. We will show that $f(k) \in D_V$ and thus that *f* is partially in $\Gamma _V$. Let $\sigma _n, \tau _n \in \Gamma _L$ be such that $\sigma _n \le f \le \tau _n$ and $\inf _{n\in \mathbb {N}}\varphi (\tau _n) = \sup _{n\in \mathbb {N}}\varphi (\sigma _n)$. Then $\inf _{n\in \mathbb {N}}(\tau _n(k) - \sigma _n(k)) \le \inf _{n\in \mathbb {N}}\varphi (\tau _n - \sigma _n) = 0$. Since $\sigma _n(k), \tau _n(k) \in D$ for all $n\in \mathbb {N}$, we have $f(k) \in D_V$.

Thus every $f\in \Gamma _{LV}$ is partially in $\Gamma _V$. Since $\Gamma _V$ is stable, by Theorem 5.5(c) we conclude that $\Gamma _{LV} \subset \Gamma _{VL}$.

### **Lemma 5.7**

Suppose that $\Gamma _{LV}$ is stable. Then every $f\in \Gamma _{LV}$ is partially in $\Gamma _V$.

### *Proof*

Let $f\in \Gamma _{LV}$ and let $\pi ,\rho \in \Gamma _L$ be such that $\pi \le f \le \rho$. Let $(A_n)_{n\in \mathbb {N}}$ be a $\varphi$-partition for both $\pi$ and $\rho$. Then $f\mathbbm {1}_{A_n} \in \Gamma _{LV}$ and $\pi \mathbbm {1}_{A_n} \le f \mathbbm {1}_{A_n} \le \rho \mathbbm {1}_{A_n}$ for all $n\in \mathbb {N}$. By Theorem 5.5(a) we conclude that $f\mathbbm {1}_{A_n}\in \Gamma _V$. $\square$

### **Theorem 5.8**

Suppose that $\Gamma _V$ and $\Gamma _{LV}$ are stable. Then $\Gamma _{LV} \subset \Gamma _{VL}=\Gamma _{LVL}$. Write $\overline{\Gamma }= \Gamma _{VL}$ and $\overline{\varphi }= \varphi _{VL}$. If $\overline{\Gamma }$ is stable, then $\overline{\Gamma }_L = \overline{\Gamma }$ and $\overline{\varphi }_L = \overline{\varphi }$. If $\overline{\Gamma }_{V}$ is stable, then $\overline{\Gamma }_V = \overline{\Gamma }$ and $\overline{\varphi }_V = \overline{\varphi }$.

In particular, if $\varphi _L(\Gamma _L)$ is mediated in *E* and $\varphi _V(\Gamma _V)$ is splitting in *E*, then $\Gamma _V$, $\Gamma _{LV}$ and $\Gamma _{VL}$ are stable (see Theorem 4.25) and thus $\Gamma _{LV}\subset \overline{\Gamma }$, $\overline{\Gamma }= \overline{\Gamma }_V = \overline{\Gamma }_L$, $\overline{\varphi }= \overline{\varphi }_V = \overline{\varphi }_L$, so $\overline{\overline{\Gamma }} = \overline{\Gamma }$ (and $\overline{ \overline{\varphi }} = \overline{\varphi })$.

### *Proof*

The inclusion $\Gamma _{LV}\subset \Gamma _{VL}$ follows by Theorem 5.5(c) and Lemma 5.7. We prove $\Gamma _{LVL}\subset \Gamma _{VL}$. For $f\in \Gamma _{LVL}^{+}$ there is a $\varphi _{LV}$-partition for *f* and since $\Gamma _{LV} \subset \Gamma _{VL}$ this is also a $\varphi _{VL}$-partition for *f*, hence there exists a $\varphi _V$-partition for *f*, i.e., $f\in \Gamma _{VL}$.

Suppose $\overline{\Gamma }$ is stable. Then $\overline{\Gamma }_{L} = (\Gamma _{VL})_L = \Gamma _{VL}= \overline{\Gamma }$ and $\overline{\varphi }_L = \overline{\varphi }$ by Theorem 4.18(a).

Suppose $\overline{\Gamma }_V$ to be stable. As $\Gamma _V$ is stable we can apply the first part of the theorem to $\Gamma _V$ instead of $\Gamma$. Indeed, $(\Gamma _V)_V$ and $(\Gamma _V)_{LV}$ are stable, since $(\Gamma _V)_V=\Gamma _V$ and $(\Gamma _V)_{LV}= \overline{\Gamma }_V$. Hence, $(\Gamma _V)_{LV} \subset (\Gamma _V)_{VL} = \Gamma _{VL}$, i.e., $\overline{\Gamma }_V \subset \overline{\Gamma }$ (and $\overline{\varphi }_V = \overline{\varphi }$).

Suppose $\varphi _L(\Gamma _L)$ is mediated in *E* and $\varphi _V(\Gamma _V)$ is splitting in *E*. Then $\Gamma _L$, $\Gamma _V$ and $\Gamma _{LV}$ are stable by Theorem 4.25(a),(b) and (c). Consequently, again by Theorem 4.25(b) $\Gamma _{VL}$ is stable. $\square$

### **Corollary 5.9**

Suppose *E* is mediated (and thus splitting), $\overline{\Gamma }= \Gamma _{VL}$. Then $\overline{\Gamma }= \overline{\Gamma }_V = \overline{\Gamma }_L$, so $\overline{\overline{\Gamma }} = \overline{\Gamma }$ (and $\overline{ \overline{\varphi }} = \overline{\varphi })$.

At the end of Sect. 5 we will show that sometimes $\Gamma _{VL} \subsetneq \Gamma _{LV}$ (Example 5.14) and sometimes $\Gamma _{LV}\subsetneq \Gamma _{VL}$ (Example 5.15). Note that this implies that $\Gamma _{VLV}$ can be strictly larger then either $\Gamma _{VL}$ or $\Gamma _{LV}$.

Theorem 5.8 raises the question whether stability of $\Gamma _V$ entails $\Gamma _{VL} \subset \Gamma _{LV}$. In general the answer is negative; see Example 5.15. In Theorem 5.10 we give conditions sufficient for the inclusion.

### **Theorem 5.10**

Suppose $\Gamma _V$ is stable. Consider these two statements.

- For every $f\in \Gamma _{VL}^{+}$ there is a $\rho$ in $\Gamma _L^{+}$ with $f\le \rho$.
- *E* satisfies:
  43 $$\begin{aligned}&\text{ If } Y_1,Y_2,\ldots \subset E \text{ are } \text{ nonempty } \text{ countable } \text{ with } \inf Y_n=0 \text{ for } \text{ all } n\in \mathbb {N}, \nonumber \\&\text{ then } \text{ there } \text{ exist } y_1\in Y_1,y_2 \in Y_2, \ldots \text{ such } \text{ that } \sum _n y_n \text{ exists } \text{ in } E. \end{aligned}$$

If (a) is satisfied, then $\Gamma _{VL}\subset \Gamma _{LV}$. (b) implies (a).

### *Proof*

If (a) is satisfied, then by Theorem 5.5(c) follows that $\Gamma _{VL} \subset \Gamma _{LV}$.

Suppose (b). Let $f\in \Gamma _{VL}^{+}$. Let $(A_n)_{n\in \mathbb {N}}$ be a $\varphi _V$-partition for *f*. For $n\in \mathbb {N}$, let $\Upsilon _n \subset \Gamma$ be a countable set with $f\mathbbm {1}_{A_n} \le \Upsilon _n$ and

44 $$\begin{aligned} \varphi _V(f\mathbbm {1}_{A_n}) = \inf \varphi (\Upsilon _n). \end{aligned}$$

We may assume $\sigma \mathbbm {1}_{A_n}= \sigma$ for all $\sigma \in \Upsilon _n$. Choose $\sigma _n \in \Upsilon _n$ for $n\in \mathbb {N}$ such that $\sum _n (\varphi (\sigma _n) - \varphi _V(f\mathbbm {1}_{A_n}))$ and thus $\sum _n \varphi (\sigma _n)$ exist in *E*. Then $\rho := \sum _{n\in \mathbb {N}} \sigma _n$ is in $\Gamma _L^{+}$ with $f \le \rho$. $\square$

### **5.11**

We will discuss examples of spaces *E* for which (43) holds.

(I) If *E* is a Banach lattice with $\sigma$-order continuous norm, then *E* satisfies (43) (one can find $y_n \in Y_n$ with $\Vert y_n\Vert \le 2^{-n})$.
(II) Let $$(X,\mathcal {A},\mu )$$ be a complete $$\sigma $$-finite measure space and assume there exists a $$g\in L^1(\mu )$$ with $$g>0$$ $$\mu $$-a.e. Then the space *E* of equivalence classes of measurable functions $$X\rightarrow \mathbb {R}$$ satisfies (43): It is sufficient to prove that if $$Z_1,Z_2,\ldots \subset E$$ are nonempty countable with $$\inf Z_n=0$$ for all $$n\in \mathbb {N}$$, then there exists $$z_1\in Z_1,z_2\in Z_2,\ldots $$ and a $$z\in E$$ such that $$z_n \le z$$ for all $$n\in \mathbb {N}$$ (for $$Z_n$$ take $$2^n Y_n)$$. One can prove that such a *z* exists by mapping the equivalence classes of measurable functions into $$L^1(\mu )$$ by the order isomorphism $$f\mapsto (\arctan \circ f)g$$.
(III) $\mathbb {R}^\mathbb {N}$ is a special case of (II), therefore satisfies (43).

### **Theorem 5.12**

Let *E* be mediated and splitting and satisfy (43) (e.g. *E* be a Banach lattice with $\sigma$-order continuous norm (Theorem 4.24), or *E* is the space mentioned in 5.11(II)). Then $\Gamma _V$ is stable and $\Gamma _{VL}=\Gamma _{LV}$, $\varphi _{VL}=\varphi _{LV}$.

### *Proof*

This is a consequence of Theorems 5.8 and 5.10. $\square$

For a Riesz space *F* and a Riesz subspace $\Gamma$ of $F^X$ we will now investigate under which conditions on $\varphi (\Gamma )$, $\varphi _L(\Gamma _L)$ and $\varphi _V(\Gamma _V)$ the spaces $\Gamma _{LV}$ and $\Gamma _{VL}$ are Riesz subspaces of $F^X$.

### **Theorem 5.13**

Suppose *F* is a Riesz space and $\Gamma$ is a Riesz subspace of $F^X$. If $\varphi (\Gamma )$ is splitting in *E* and $\varphi _L(\Gamma _L)$ is mediated in *E*, then $\Gamma _{LV}$ is a Riesz subspace of $F^X$. If $\varphi (\Gamma )$ is mediated in *E* and $\varphi _V(\Gamma _V)$ is splitting in *E*, then $\Gamma _{VL}$ is a Riesz subspace of $F^X$.

In particular, if *E* is mediated (and thus splitting), then both $\Gamma _{LV}$ and $\Gamma _{VL}$ are Riesz subspaces of $F^X$.

### *Proof*

Note first that if $\varphi (\Gamma )$ is mediated in *E*, then $\Gamma _V$ is stable by Theorem 4.25(b). For a proof, combine Theorem 4.32 and Corollary 3.10. $\square$

The next example illustrates that $\Gamma _{LV}$ is not always included in $\Gamma _{VL}$ (given that $\Gamma _V$ is stable) even if *E* and *F* are Riesz spaces and $\Gamma , \Gamma _{LV}, \Gamma _{VL}$ Riesz subspaces of $F^X$.

### *Example 5.14*

[$\Gamma _{VL} \subsetneq \Gamma _{LV} = \Gamma _{VLV}$] For an element $b= (\beta _1,\beta _2,\ldots )$ of $\mathbb {R}^\mathbb {N}$ we write $b = \sum _{n\in \mathbb {N}} \beta _n e_n$.

Consider $X=\{0,1,2,\ldots \}$ and $\mathcal {I}= \mathcal {P}(X)$. Let $E= c$, $F= \mathbb {R}^\mathbb {N}$, $\Omega = F^X$. We view the elements of $\Omega$ as sequences $(a,b_1,b_2,\ldots )$ with $a,b_1,b_2,\ldots \in \mathbb {R}^\mathbb {N}$.

Define sets $\Gamma \subset \Theta \subset \Omega$ and a map $\Phi : \Theta \rightarrow \mathbb {R}^\mathbb {N}$ by

45 $$\begin{aligned}&\Theta = \{ (a,\beta _1e_1,\beta _2 e_2, \ldots ) : a\in c, \beta _1,\beta _2,\ldots \in \mathbb {R}\}, \end{aligned}$$

46 $$\begin{aligned}&\Phi (a,\beta _1e_1,\beta _2e_2,\ldots ) = a + \sum _{n\in \mathbb {N}} \beta _n e_n \quad (a\in c, \ \beta _1,\beta _2,\ldots \in \mathbb {R}), \end{aligned}$$

47 $$\begin{aligned}&\Gamma = \{ (a, \beta _1 e_1, \beta _2 e_2, \ldots ) : a\in c, \ (\beta _1,\beta _2,\ldots ) \in c_{00} \}. \end{aligned}$$

Then $\Phi (\Gamma ) = c = E$; let $\varphi = \Phi |_{\Gamma }$. From the definition it is easy to see that $\Gamma$ is stable and $\varphi$ is laterally extendable. We leave it to the reader to verify that $\Gamma _V= \Gamma$,

48 $$\begin{aligned} \Gamma _L = \{ (a,\ \beta _1e_1, \beta _2 e_2, \ldots ): a\in c, \ (\beta _1,\beta _2,\ldots ) \in c\} \end{aligned}$$

and $$\varphi _L= \Phi $$ on $$\Gamma _L$$.

It follows that $\Gamma _V$ is stable and $\Gamma _{VL}= \Gamma _L \subset \Gamma _{LV} = \Gamma _{VLV}$. We prove $\Gamma _{VL} \ne \Gamma _{LV}$. To this end, define $h\in \Omega$ by

49 $$\begin{aligned} {\left\{ \begin{array}{ll} h(n)= (-1)^n e_n \quad (n=1,2,\ldots ), \\ h(0)= - \sum _{n\in \mathbb {N}} h(n) = - \sum _{n\in \mathbb {N}} (-1)^n e_n. \end{array}\right. } \end{aligned}$$

As $h(0) \notin c$ we have $h\mathbbm {1}_{\{0\}} \notin \Gamma$; in particular, *h* is not partially in $\Gamma$, so $h\notin \Gamma _L = \Gamma _{VL}$. It remains to prove $h\in \Gamma _{LV}$.

For $$k\in \mathbb {N}$$, define $$\tau _k, \sigma _k : X \rightarrow \mathbb {R}^\mathbb {N}$$:

50 $$\begin{aligned}&{\left\{ \begin{array}{ll} \tau _k(0)= - \sum _{n=1}^k (-1)^n e_n + \sum _{n=k+1}^\infty e_n, \\ \tau _k(n)= h(n) = (-1)^n e_n &{} (n=1,\ldots ,k), \\ \tau _k(n)= e_n &{}(n=k+1,k+2,\ldots ), \end{array}\right. } \end{aligned}$$

51 $$\begin{aligned}&{\left\{ \begin{array}{ll} \sigma _k(0)= - \sum _{n=1}^k (-1)^n e_n - \sum _{n=k+1}^\infty e_n, \\ \sigma _k(n)= h(n) = (-1)^n e_n &{} (n=1,\ldots ,k), \\ \sigma _k(n)= - e_n &{} (n=k+1,k+2,\ldots ). \end{array}\right. } \end{aligned}$$

Then $\tau _k, \sigma _k\in \Gamma _L$, $\tau _k \ge h \ge \sigma _k$, $\varphi _L(\tau _k) = \Phi (\tau _k) = 2 \sum _{n>k} e_n$, $\varphi _L(\sigma _k) = - 2 \sum _{n>k} e_n$, so $\inf _{k\in \mathbb {N}}\varphi _L(\tau _k) = \sup _{k\in \mathbb {N}}\varphi _L(\sigma _k) =0$, and $h\in \Gamma _{LV}$.

The next example illustrates that $\Gamma _{VL}$ is not always included in $\Gamma _{LV}$; it provides an example of an $f\in \Gamma _{VL}^{+}$ for which there exist no $\rho \in \Gamma _L^{+}$ with $f \le \rho$ (see Theorem 5.5(c)).

### *Example 5.15*

[$\Gamma _{LV} \subsetneq \Gamma _{VL}$] Let $E= C[0,1]$ and let $D \subset C[0,1]$ be the set of polynomials of degree $\le 2$. The set *D* is order dense^7^ in *C*[0, 1] (see [11, Example4.4]). Hence, for all $f\in E$ there exist $(g_n)_{n\in \mathbb {N}},(h_n)_{n\in \mathbb {N}}$ in *D* with $f= \inf _{n\in \mathbb {N}}g_n = \sup _{n\in \mathbb {N}}h_n$. Therefore *E* is the vertical extension of *D* with respect to the inclusion map $D\rightarrow E$.

Take $X = \mathbb {N}$, $\mathcal {I}= \mathcal {P}(\mathbb {N}), F=E=C[0,1], \Gamma = c_{00}[D] \subset F^\mathbb {N}=E^\mathbb {N}$ and let $\varphi : \Gamma \rightarrow E$ be given by $\varphi (f) = \sum _{n\in \mathbb {N}} f(n)$. Since this situation is the same as in Example 5.6 with $D_V=E$, we have $\Gamma _V = c_{00}[E]$ and $\Gamma _{LV} \subset \Gamma _{VL}$.

Furthermore (see 4.6)

52 $$\begin{aligned} \Gamma _L^{+}&= \left\{ f \in (D^{+})^\mathbb {N}: \sum _n f(n) \text{ exists } \text{ in } E\right\} , \end{aligned}$$

53 $$\begin{aligned} \Gamma _{VL}^{+}&= \left\{ f \in (E^{+})^\mathbb {N}: \sum _n f(n) \text{ exists } \text{ in } E\right\} . \end{aligned}$$

**Fig. 1:**
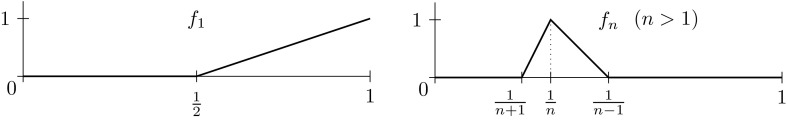
Graph of $$f_n$$

We construct an $f\in \Gamma _{VL}^{+}$ that is not in $\Gamma _{LV}$. For $n\in \mathbb {N}$ let $f_n$ be the ‘tent’ function defined by (Fig. 1)

54 $$\begin{aligned}&f_n(0)=0; \quad f_n(\tfrac{1}{n})=1; \quad f_n(\tfrac{1}{i})=0 \quad \text{ if } i\in \mathbb {N}, i\ne n; \nonumber \\&f_n \text{ is } \text{ affine } \text{ on } \text{ the } \text{ interval } [\tfrac{1}{1+i},\tfrac{1}{i}] \text{ for } \text{ all } i \in \mathbb {N}. \end{aligned}$$

Then $\sum _{n=1}^\infty f_n = \mathbbm {1}_{(0,1]}$ pointwise, so $\sum _n f_n =\mathbbm {1}$ in *C*[0, 1]. Hence $f= (f_1,f_2,f_3,\ldots )\in \Gamma _{VL}^{+}$.

We will prove that $f\notin \Gamma _{LV}$; by showing there exists no $\rho \in \Gamma _L$ for which $f \le \rho$.

Suppose $\rho \in \Gamma _L$ and $f\le \rho$. Then $\rho = (\rho _1, \rho _2, \ldots )$ where $\rho _1,\rho _2,\ldots$ are elements of $D^{+}$ and $j = \sum _n \rho _n$ exists in $E= C[0,1]$. Let *M* be the largest value of *j*. Every $\rho _n$ is a quadratic function that maps [0, 1] into [0, *M*]. Consequently (see the postscript)

55 $$\begin{aligned} |\rho _n(x) - \rho _n(y) | \le 4 M |x-y| \quad (x,y\in [0,1], n\in \mathbb {N}). \end{aligned}$$

In particular, $\rho _n(0) \ge \rho _n( \frac{1}{n} ) - 4M \frac{1}{n} \ge f_n(\frac{1}{n}) - 4M \frac{1}{n}= 1 - 4M \frac{1}{n} \ge \frac{1}{2}$ for $n\ge 8M$. As $j(0) \ge \sum _{n\ge N} \rho _n(0)$ for all $N\in \mathbb {N}$, this is a contradiction.

*Postscript* Let $h : x \mapsto a x^2 +bx +c$ be a quadratic function on [0, 1] and $0 \le h(x) \le M$ for all *x*; we prove $|h'(x)|\le 4M$ for all $x\in [0,1]$. Since the derivative is either decreasing or increasing, we have $|h'(x)| \le \max \{|h'(0)|, |h'(1)|\}$. Now $h'(0)=b = 4h(\tfrac{1}{2}) -h(1) -3 h(0)$ and $h'(1) = 2a+b = 3h(1) + h(0) - 4h(\tfrac{1}{2})$. Since $|h(x)-h(y)|\le M$ for all $x,y\in [0,1]$, we get the bounds $|h'(0)|\le 4M$ and $|h'(1)|\le 4M$ as desired.

### **5.16**

Observe that $\Gamma _{VL}$ in Example 5.15 is not stable since $(f_1,0,f_3,0,\ldots )\notin \Gamma _{VL}$.

## Embedding *E* in a (slightly) larger space

**In this section** $\mathbf {E,F,X},\varvec{\mathcal {I},\Gamma ,\varphi }$ **are as in Sect**. 4.

Suppose $E^\bullet$ is another partially ordered vector space and $E \subset E^\bullet$. Consider $\varphi ^\bullet : \Gamma \rightarrow E^\bullet$, where $\varphi ^\bullet (f) = \varphi (f)$ for $f\in \Gamma$.

Write $\Gamma _{V}^\bullet$ for the vertical extension of $\Gamma$ with respect to $\varphi ^\bullet$. If $\varphi ^\bullet$ is laterally extendable, write $\Gamma ^\bullet _L$ for the lateral extension of $\Gamma$ with respect to $\varphi ^\bullet$, $\Gamma _{LV}^\bullet$ for the vertical extension of $\Gamma _L^\bullet$ with respect to $\varphi _L^\bullet$. Similarly, if $\Gamma _V^\bullet$ is stable, we introduce the notations $\Gamma _{VL}^\bullet$ and $\Gamma _{VLV}^\bullet$.

It is not generally the case that $\Gamma _V \subset \Gamma _V^\bullet$ or $\Gamma _L \subset \Gamma _L^\bullet$, but a natural restriction on $E^\bullet$ helps; see Theorem 6.2.

For $E^\bullet$ we can choose to be a Dedekind complete Riesz space in which countable suprema of *E* are preserved, in case *E* is Archimedean and directed (see 6.3). In this situation, in some sense, $\Gamma _{VL}^\bullet$ is the largest extension one can obtain.

### **Definition 6.1**

Let *D* be a subspace of a partially ordered vector space *P*. Then we say that *countable suprema in* *D* *are preserved in* *P* if the following implication holds for all $a\in D$ and all countable $A\subset D$

56 $$\begin{aligned} A \text{ has } \text{ supremum } a \text{ in } D \Longrightarrow A \text{ has } \text{ supremum } a \text{ in } P. \end{aligned}$$

Note that the reverse implication holds always.

The following theorem is a natural consequence.

### **Theorem 6.2**

Suppose that countable suprema in *E* are preserved in $E^\bullet$. Then $\varphi ^\bullet$ is laterally extendable and

57 $$\begin{aligned}&f\in \Gamma _V \iff f\in \Gamma _{V}^\bullet \text{ and } \varphi ^\bullet _V(f) \in E, \end{aligned}$$

58 $$\begin{aligned}&f\in \Gamma _L \iff f\in \Gamma _{L}^\bullet \text{ and } \varphi ^\bullet _L(f) \in E, \end{aligned}$$

59 $$\begin{aligned}&\varphi _V^\bullet (f) = \varphi _V(f) \text{ for } f\in \Gamma _V, \quad \varphi _L^\bullet (f) = \varphi _L(f) \text{ for } f\in \Gamma _L, \end{aligned}$$

60 $$\begin{aligned}&\Gamma _{LV} \subset \Gamma _{LV}^\bullet , \quad \varphi _{LV}^\bullet (f) = \varphi _{LV}(f) \text{ for } f\in \Gamma _{LV}. \end{aligned}$$

Suppose $\Gamma _V$ and $\Gamma _V^\bullet$ are stable. Then

61 $$\begin{aligned}&\Gamma _{VL} \subset \Gamma _{VL}^\bullet , \quad \varphi _{VL}^\bullet (f) = \varphi _{VL}(f) \text{ for } f\in \Gamma _{VL}, \end{aligned}$$

62 $$\begin{aligned}&\Gamma _{VLV} \subset \Gamma _{VLV}^\bullet , \quad \varphi _{VLV}^\bullet (f) = \varphi _{VLV}(f) \text{ for } f\in \Gamma _{VLV}. \end{aligned}$$

### **6.3**

Under the assumptions made in Sect. 4 $\Gamma$ is directed, thus so are $\Gamma _L$, $\Gamma _V$ (see 3.11) and $\Gamma _{LV}$ (etc.). Hence $\varphi _V(\Gamma _V)$, $\varphi _L(\Gamma _L)$, $\varphi _{LV}(\Gamma _{LV})$ (etc.) are all subsets of $E^{+} - E^{+}$. For this reason we may assume that *E* itself is directed.

Then under the (rather general) assumption that *E* is also Archimedean (see Definition 3.19), *E* can be embedded in a Dedekind complete Riesz space such that suprema and infima in *E* are preserved, as we state in Theorem 6.4.

Consequently, choosing such a Dedekind complete Riesz space for $E^\bullet$ one has the following: $\Gamma ^\bullet _V$, $\Gamma ^\bullet _{LV}$, $\Gamma ^\bullet _{VL}$, $\Gamma _{VLV}^\bullet$ are stable and $\Gamma ^\bullet _{LV} \subset \Gamma ^\bullet _{VL}=: \overline{\Gamma }^\bullet$, $\overline{\Gamma }^\bullet _L = \overline{\Gamma }^\bullet _V = \overline{\Gamma }^\bullet$ and $\overline{\varphi }^\bullet _L = \overline{\varphi }^\bullet _V = \overline{\varphi }^\bullet$, where $\overline{\varphi }^\bullet :=\varphi _{VL}^\bullet$ (see 5.8). Moreover, one has (60) and if $\Gamma _V$ is stable; (61) and (62). For this reason one may consider $\overline{\Gamma }^\bullet$ and $\overline{\varphi }^\bullet$ instead of $\Gamma _{LV}$ and $\varphi _{LV}$, instead of $\Gamma _{LV}^\bullet$ and $\varphi ^\bullet _{LV}$ or instead of $\Gamma _{VLV}$ and $\varphi _{VLV}$, indeed $\overline{\Gamma }^\bullet$ contains all of the other extensions and $\overline{\varphi }^\bullet$ agrees with all integrals.

### **Theorem 6.4**

[8, Chapter 4,Theorem 1.19] Let *E* be an Archimedean directed partially ordered vector space. Then *E* can be embedded in a Dedekind complete Riesz space $\hat{E}$:

There exists an injective linear $\gamma : E \rightarrow \hat{E}$ for which

- $$a \ge 0 \iff \gamma (a) \ge 0$$,
- $\gamma (E)$ is order dense in $\hat{E}$ (for the definition of order dense see the seventh footnote).

Consequently, suprema in $\gamma (E)$ are preserved in $\hat{E}$.

## Integration for functions with values in $\mathbb {R}$

**In this section** $\mathbf {(X,}\varvec{\mathcal {A},\mu }\mathbf {)}$ **is a complete** $\varvec{\sigma }$ **-finite measure space and** $\mathbf {E=F=}\,\,\varvec{\mathbb {R}}$.

We write *S* for the vector space of simple functions from *X* to $\mathbb {R}$ (see 4.33). Since $\mathbb {R}$ is a Banach lattice with $\sigma$-order continuous norm, $S_V$ is stable and $S_{LV}=S_{VL}$, $\varphi _{LV}=\varphi _{VL}$ (by Theorem 5.12). We write $\overline{S}= S_{VL}$ and $\overline{\varphi }= \varphi _{VL}$.

### **Theorem 7.1**

$\overline{S}= \mathcal {L}^1(\mu )$ and $\overline{\varphi }(f)= \int f {{\mathrm{\, \mathrm {d}}}}\mu$ for all $f\in \overline{S}$.

### *Proof*

We prove that $S_{VL}^{+} \subset \mathcal {L}^1(\mu )^{+} \subset S_{LV}^{+}$ and that $\varphi _{LV}(f) = \int f {{\mathrm{\, \mathrm {d}}}}\mu$ for all $f\in \mathcal {L}^{+}(\mu )$.

$S_V$ consists of the bounded integrable functions *f* for which $\{x\in X: f(x) \ne 0\}$ has finite measure. By monotone convergence, we have $f\in \mathcal {L}^1(\mu )$ for every $f\in S_{VL}^{+}$.

Conversely, let $f\in \mathcal {L}^1(\mu )^{+}$; we prove $f\in S_{LV}^{+}$ and $\varphi _{LV}(f) = \int f {{\mathrm{\, \mathrm {d}}}}\mu$. Let $t\in (1,\infty )$. For $n\in \mathbb {Z}$, put $A_n = \{x\in X: t^n \le f(x) < t^{n+1} \}$. Then $(A_n)_{n\in \mathbb {Z}}$ forms a partition. Define $g: = \sum _{n\in \mathbb {Z}} t^n \mathbbm {1}_{A_n}$ and $h: = tg$; then $g\le f \le h$. Since63we have $g\in S_L$ and $\varphi _L(g) \le \int f {{\mathrm{\, \mathrm {d}}}}\mu$. Also, $h= tg \in S_L$, and $\varphi _L(h) - \varphi _L(g) = (t-1) \varphi _L(g) \le (t-1) \int f {{\mathrm{\, \mathrm {d}}}}\mu$. By this and Lemma 3.7 it follows that $f\in S_{LV}$ and $\varphi _{LV}(f) = \int f {{\mathrm{\, \mathrm {d}}}}\mu$. $\square$

## Extensions of integrals on simple functions

**In this section** $\mathbf{E}$ **is a directed partially ordered vector space,** $\mathbf {(X,}\varvec{\mathcal {A},\mu }\mathbf {)}$ **is a complete** $\varvec{\sigma }$ **-finite measure space and** $\varvec{\mathcal {I}},\mathbf {S},\varvec{\varphi }$ **are as in** 4.33 ($\mathbf{F=E}$)

In 8.1–8.8 for *f* in $S_{LV}$ or $S_{VL}$ we discuss the relation between *f* being almost everywhere equal to zero and *f* having integral zero (i.e., either $\varphi _{LV}(f)=0$ or $\varphi _{VL}(f)=0$).

In 8.9 we show that under some conditions a function in $S_V$ multiplied with an integrable function with values in $\mathbb {R}$ is a function in $S_{LV}$.

In 8.11–8.13 we investigate the relation between the “*LV*”-extension on simple functions with respect to $\mu$ and $\nu$, where $\nu = h \mu$ for some measurable $h: X \rightarrow [0,\infty )$.

In 8.14 we discuss the relation between the “*LV*”-extension simple functions with values in *E* or in another partially ordered vector space *F*, when one makes the composition of a function in the extension with a $\sigma$-order continuous linear map $E\rightarrow F$.

In 8.15–8.17 we will prove that under certain conditions on *X* the function $x\mapsto F(x,\cdot )$ is in $S_V$ for all $F\in C(X\times T)$ and we relate that to convolution of certain finite measures with continuous functions on a topological group.

### **Theorem 8.1**

Let $f: X \rightarrow E$ and $f=0$ a.e. If $f\in S_{LV}$, then $\varphi _{LV}(f)=0$. If $S_V$ is stable and $f\in S_{VLV}$, then $\varphi _{VLV}(f)=0$.

### *Proof*

Let $B= \{x\in X: f(x) \ne 0\}$. Then $B\in \mathcal {A}$ and $\mu (B)=0$.

(I) Assume $f\in S_V$. Choose $\sigma , \tau \in S$ with $\sigma \le f\le \tau$. Then $\sigma \mathbbm {1}_B, \tau \mathbbm {1}_B \in S$, $\sigma \mathbbm {1}_B \le f \le \tau \mathbbm {1}_B$, and $\varphi (\sigma \mathbbm {1}_B) = \varphi (\tau \mathbbm {1}_B) =0$. Hence $\varphi _V(f) =0$.
(II) Suppose $\sigma \in S_L^{+}$ and $(A_n)_{n\in \mathbb {N}}$ is a $\varphi$-partition for $\sigma$. Then $\sigma \mathbbm {1}_{A_n \cap B} \in S^{+}$ for all $n\in \mathbb {N}$ and $\sum _n \varphi (\sigma \mathbbm {1}_{A_n\cap B})=0$, i.e., $\sigma \mathbbm {1}_B \in S_L^{+}$ with $\varphi _L(\sigma \mathbbm {1}_B)=0$. In particular, if $f\in S_L$ then $\varphi _L(f)=0$.
(III) Assume $f\in S_{LV}$. With (II) one can repeat the argument of (I) with *S* replaced by $S_L$ and conclude $\varphi _{LV}(f)=0$.
(IV) Suppose $S_V$ is stable and $f\in S_{VLV}$. One can repeat the argument in (III) with *S* replaced by $S_V$ and conclude $\varphi _{VLV}(f)=0$.

$$\square $$

### **Definition 8.2**

A subset $D\subset E$ is called *order bounded* if there are $a,b\in E$ for which $a\le D \le b$.

### **Theorem 8.3**

Let $f\in S_{LV}$ or (assuming $S_V$ is stable) $f\in S_{VLV}$. Then there exists a partition $(A_n)_{n\in \mathbb {N}}$ such that each set $f(A_n)$ is order bounded.

### *Proof*

There exists a partition $(A_n)_{n\in \mathbb {N}}$ such that for all $n\in \mathbb {N}$ there exist $h_n,g_n\in S$ for which $h_n \le f\mathbbm {1}_{A_n} \le g_n$. Choose $a_n,b_n \in E$ for which $a_n \le h_n(x)$ and $g_n(x)\le b_n$ for all $x\in X$. Then $a_n \le f(x) \le b_n$ for $n\in \mathbb {N}$, $x\in A_n$. $\square$

### **Theorem 8.4**

Let $f: X \rightarrow E$ and $f=0$ a.e. Suppose there exists a partition $(A_n)_{n\in \mathbb {N}}$ such that for every $n\in \mathbb {N}$ the subset $f(A_n)$ of *E* is order bounded. Then $f\in S_{LV}$ and if $S_V$ is stable then also $f\in \Gamma _{VL}$.

### *Proof*

Choose $a_1,a_2,\ldots$ and $b_1,b_2,\ldots$ in *E* such that

64 $$\begin{aligned} a_n \le f(x) \le b_n \quad (n\in \mathbb {N}, x\in A_n). \end{aligned}$$

Let $B=\{x\in X: f(x) \ne 0\}$. Then $B\in \mathcal {A}$ and $\mu (B)=0$. Hence $g:= \sum _{n\in \mathbb {N}} a_n \mathbbm {1}_{A_n\cap B}$ and $h:= \sum _{n\in \mathbb {N}} b_n \mathbbm {1}_{A_n \cap B}$ are elements of $S_L$ with $\varphi (g)=0$ and $\varphi _L(h)=0$. As $g \le f \le h$, we get $f\in S_{LV}$ and if $S_V$ is stable also $f\in S_{VL}$. $\square$

For a real valued function $f: X \rightarrow \mathbb {R}$ with $f\ge 0$ and $\int f {{\mathrm{\, \mathrm {d}}}}\mu =0$ we have $f=0$ a.e. We will give an example of a $f\in S_V^{+}$ with $\varphi _V(f) =0$ but which is nowhere zero (Example 8.8). On the positive side, in Theorem 8.7 we show that $f=0$ a.e. if $f\in S_{LV}^{+}$ and $\varphi _{LV}(f)=0$ provided that *E* satisfies a certain separability condition.

### **Definition 8.5**

We call a subset *D* of $E^{+}{\setminus } \{0\}$ *pervasive* 8 in *E* if for all $a\in E$ with $a>0$ there exists a $d\in D$ such that $0<d\le a$. We say that *E* *possesses a pervasive subset* if there exists a pervasive $D\subset E^{+}{\setminus } \{0\}$.

### *Example 8.6*

The Riesz spaces $\mathbb {R}^\mathbb {N}, \ell ^\infty , c, c_0, \ell ^1$ and $c_{00}$ possess countable pervasive subsets. Indeed, in each of them the set $\{ \lambda e_n: \lambda \in \mathbb {Q}^{+}, \lambda >0, n\in \mathbb {N}\}$ is pervasive.

If $\mathcal {X}$ is a completely regular topological space, then $C(\mathcal {X})$ has a countable pervasive subset if and only if $\mathcal {X}$ has a countable base. (If $D\subset E^{+}{\setminus } \{0\}$ is countable and pervasive, then $\mathfrak {U}= \{ f^{-1}(0,\infty ): f\in D\}$ is a countable base; vise versa if $\mathfrak {U}$ is a countable base then with choosing an $f_U$ in $C(X)^{+}$ for each $U\in \mathfrak {U}$ with $f_U=0$ on $U^c$ and $f_U(x)=1$ for some $x\in U$, the set $D= \{ \varepsilon f_U : \varepsilon \in \mathbb {Q}, \varepsilon >0, U\in \mathfrak {U}\}$ is pervasive.)

$L^1(\lambda )$ and $L^\infty (\lambda )$ do not possess countable pervasive subsets, considering the Lebesgue measure space $(\mathbb {R},\mathcal {M},\lambda )$. (Suppose one of them does. Then one can prove the existence of non-negligible measurable sets $A_1,A_2,\ldots \in \mathcal {M}$ such that every non-negligible measurable set contains an $A_n$, whereas $\lambda (A_n) <2^{-n}$ for all $n\in \mathbb {N}$. Putting $C= \mathbb {R}{\setminus } \bigcup _{n\in \mathbb {N}} A_n$ we have a non-negligible measurable set that contains no $A_n$: a contradiction.)

### **Theorem 8.7**

Let *E* possess a countable pervasive subset *D*. Let $f\in S_{LV}$. Let $\Lambda , \Upsilon \subset S_L$ be countable sets such that $\Lambda \le f \le \Upsilon$ and $\sup \varphi _L(\Lambda ) = \inf \varphi _L(\Upsilon )$. Then for almost all $x\in X$

65 $$\begin{aligned} \sup _{g\in \Lambda } g(x) = f(x) = \inf _{h\in \Upsilon } h(x). \end{aligned}$$

Consequently, if $f\in S_{LV}^{+}$ and $\varphi _{LV} (f) =0$, then $f=0$ a.e. (However, see Example 8.8.)

### *Proof*

(I) First, as a special case (namely $f=0$), let $(\tau _n)_{n\in \mathbb {N}}$ be a sequence in $S_L$ with $\tau _n \ge 0$ for all $n\in \mathbb {N}$ and $\inf _{n\in \mathbb {N}} \varphi _L( \tau _n )=0$. We prove that $\inf _{n\in \mathbb {N}}\tau _n(x) = 0$ for almost all $x\in X$, by proving that $\mu (A)=0$, where *A* is the complement of the set $\{x\in X: \inf _{n\in \mathbb {N}}\tau _n(x)=0\}$. Indeed, for this *A* we have
  66 $$\begin{aligned} A= \bigcup _{d\in D} A_d, \quad \text{ with } \quad A_d = \bigcap _{n\in N} \{x\in X: d\le \tau _n(x)\}. \end{aligned}$$
  Note that for all $n\in \mathbb {N}$ and $d\in D$ the set $\{x\in X: d\le \tau _n(x)\}$ is measurable. Furthermore, for all $d\in D$ we have:
  67 $$\begin{aligned} d \mu (A_d ) = \varphi ( d \mathbbm {1}_{ A_d }) \le \varphi _L(\tau _n) \quad (n\in \mathbb {N}). \end{aligned}$$
  Hence $\mu ( A_d)=0$ for all $d\in D$ and thus $\mu (A) =0$.
(II) Suppose that $\Lambda , \Upsilon \subset \Gamma _L$ are countable sets such that $\Lambda \le f \le \Upsilon$, $\sup \varphi _L(\Lambda ) = \inf \varphi _L(\Upsilon )$. Then $\inf \varphi _L(\Upsilon - \Lambda ) =0$, so by (I) $\inf _{g\in \Upsilon , h\in \Lambda } (g(x) - h(x)) = 0$ for almost all $x\in X$.

$$\square $$

### *Example 8.8*

We give an example of a $f\in S_V^{+}$ with $\varphi _V(f) =0$, where $f\ne 0$ everywhere. Let $([0,1), \mathcal {M}, \lambda )$ be the Lebesgue measure space with underlying set [0, 1). Let $E= \ell ^\infty ([0,1))$ (see Sect. 2). Let $f: \mathbb {R}\rightarrow E^{+}$ be defined by $f(t) = \mathbbm {1}_{\{t\}}$ for $t\in [0,1)$. Note that *f* is not partially in *S*. We will show $f\in S_{V}$. For $n\in \mathbb {N}$ make $\tau _n \in S$:

68 $$\begin{aligned} \tau _n (t) = \mathbbm {1}_{[\frac{i-1}{n},\frac{i}{n})} \quad \text{ if } \ i \in \{1,\ldots ,n\}, t\in [\tfrac{i-1}{n},\tfrac{i}{n}). \end{aligned}$$

Then $\varphi (\tau _n) = \frac{1}{n} \mathbbm {1}_{[0,1)}$ and $0 \le f \le \tau _n$ for $n\in \mathbb {N}$, so $f\in S_V$ and $\varphi _V(f) =0$. But $f(t) \ne 0$ for all *t*.

### **Theorem 8.9**

Let *E* be Archimedean and mediated. Let $f: X \rightarrow E$ and $g: X \rightarrow \mathbb {R}$. We write *gf* for the function $x\mapsto g(x) f(x)$. Then

- $f\in S_{V}$ and *g* is bounded and measurable $\Longrightarrow$ $gf\in S_{V}$.
- *f* is partially in $S_V$ and *g* is measurable $\Longrightarrow$ *gf* is partially in $S_V$.
- $$f\in S_V$$ and $$g\in \mathcal {L}^1(\mu )$$ $$\Longrightarrow $$ $$gf\in S_{LV}$$.
- $f\in S_{VL}$ and *g* is bounded and measurable $\Longrightarrow$ $gf\in S_{VL}$.
- $f\in S_{VL}$, *f*(*X*) is order bounded and $g\in \mathcal {L}^1(\mu )$ $\Longrightarrow$ $gf\in S_{VL}$.

### *Proof*

*E* is splitting (see 4.23(b)).

- is a consequence of Theorem 3.21(a) (see also Remark 3.22).
- Let $(A_n)_{n\in \mathbb {N}}$ be a partition such that $f\mathbbm {1}_{A_n} \in S_V$ and $g\mathbbm {1}_{A_n}$ is bounded for all $n\in \mathbb {N}$. By (a) every $gf\mathbbm {1}_{A_n}$ lies in $S_V$. Then *gf* is partially in $S_V$.
- Assume $f\ge 0$ and $g \ge 0$. Choose (see the proof of Theorem 7.1) a partition $(A_n)_{n\in \mathbb {N}}$ and numbers $\lambda _1,\lambda _2,\ldots$ in $[0,\infty )$ with
  69 $$\begin{aligned} \tau := \sum _{n\in \mathbb {N}}\lambda _n \mathbbm {1}_{A_n} \ge g, \quad \sum _{n\in \mathbb {N}}\lambda _n \mu (A_n) <\infty . \end{aligned}$$
  Then $\tau s \in S_{L}$ for all $s\in S$. Choose $s\in S$ with $s\ge f$. Then $0 \le gf \le \tau s$. From Theorem 5.5(e) and (b) it follows that $gf\in S_{LV}$.
- Assume $f\ge 0$ and $0\le g \le \mathbbm {1}$. Using (b), choose a partition $(A_n)_{n\in \mathbb {N}}$ with $f\mathbbm {1}_{A_n}\in S_V$ and $gf\mathbbm {1}_{A_n} \in S_V$ for all $n\in \mathbb {N}$. Then
  70 $$\begin{aligned}&0 \le \varphi _V(gf\mathbbm {1}_{A_n}) \le \varphi _V(f\mathbbm {1}_{A_n}) \quad (n\in \mathbb {N}). \end{aligned}$$
  Since $\sum _n \varphi _V(f \mathbbm {1}_{A_n})$ exists and *E* is splitting, $\sum _n \varphi _V(gf\mathbbm {1}_{A_n})$ exists.
- Assume $f\ge 0$ and $g \ge 0$. Choose $a\in E^{+}$ with $f(x) \le a$ for all $x\in X$. Choose a partition $(A_n)_{n\in \mathbb {N}}$ and $\lambda _1,\lambda _2,\ldots \in [0,\infty )$ with
  71 $$\begin{aligned}&gf\mathbbm {1}_{A_n} \in S_V \qquad (n\in \mathbb {N}), \end{aligned}$$
  72 $$\begin{aligned}&g \le \sum _{n\in \mathbb {N}}\lambda {\mathbbm {1}}_{{A_{n}}}, \qquad {\sum _{n\in \mathbb {N}}} \lambda _{n} \mu (A_{n}) <\infty \quad \mathrm{(see\,the\,proof\,of\,Theorem\,7.1)}. \end{aligned}$$
  Then
  73 $$\begin{aligned}&g f \mathbbm {1}_{A_n} \le \lambda _n a \mathbbm {1}_{A_n} \quad (n\in \mathbb {N}), \end{aligned}$$
  74 $$\begin{aligned}&\varphi _V(\lambda _n a \mathbbm {1}_{A_n}) = \varphi (\lambda _n a \mathbbm {1}_{A_n}) = \lambda _n \mu (A_n) a \quad (n\in \mathbb {N}), \end{aligned}$$
  so $\sum _n \varphi _V(\lambda _n a \mathbbm {1}_{A_n})$ exists and so does $\sum _n \varphi _V(gf \mathbbm {1}_{A_n})$.

$$\square $$

### **8.10**

In Lemma 8.11, Theorems 8.12 and 8.13 we investigate the relation between the extensions $S_{LV}$ generated by two different measures, namely $\mu$ and $h\mu$ for a measurable function $h: X \rightarrow [0,\infty )$.

Note that for such a function *h* and all $s\in (1,\infty )$ there exists a $j: X \rightarrow [0,\infty )$ that is partially in the space of simple functions $X \rightarrow [0,\infty )$, i.e., $j= \sum _{n\in \mathbb {N}}\alpha _n \mathbbm {1}_{A_n}$ for a partition $(A_n)_{n\in \mathbb {N}}$ and $(\alpha _n)_{n\in \mathbb {N}}$ in $[0,\infty )$ (or in the language of  3.16 *j* is partially in $[\mathcal {A}]$) for which $j \le h \le sj$. In the following (8.11, 8.12 and 8.13) we will write $\mathcal {I}^\mu$, $S^\mu$ and $\varphi ^\mu$ instead of $\mathcal {I}$, *S* and $\varphi$ and, similarly for another measure $\nu$ on $(X,\mathcal {A})$, we write $\mathcal {I}^\nu ,S^\nu$ and $\varphi ^\nu$ according to 4.33 with $\nu$ instead of $\mu$.

### **Lemma 8.11**

Suppose *E* is splitting. Let $h: X \rightarrow [0,\infty )$ be measurable, $\nu := h \mu$. Let $s\in (1,\infty )$ and let $j: X \rightarrow [0,\infty )$ be partially in $[\mathcal {A}]$ and such that $j\le h \le sj$. Let $f\in S_L^{\nu +}$. Then $jf\in S^\mu _L$ and $\varphi _L^\mu (jf) \le \varphi _L^\nu (f) \le s \varphi _L^\mu (jf)$.

### *Proof*

Assume $(A_n)_{n\in \mathbb {N}}$ is a partition for *j* and a $\varphi ^\mu$-partition for *f* (so $(A_n)_{n\in \mathbb {N}}$ is in $\mathcal {I}^\nu \cap \mathcal {I}^\mu$, i.e., $\mu (A_n), \nu (A_n)<\infty$ for all $n\in \mathbb {N}$). Choose $(\alpha _n)_{n\in \mathbb {N}}$ in $[0,\infty )$ and $(b_n)_{n\in \mathbb {N}}$ in $E^{+}$ such that

75 $$\begin{aligned} j = \sum _{n\in \mathbb {N}}\alpha _n \mathbbm {1}_{A_n}, \quad f = \sum _{n\in \mathbb {N}}b_n \mathbbm {1}_{A_n}. \end{aligned}$$

Then $jf = \sum _{n\in \mathbb {N}}\alpha _n b_n \mathbbm {1}_{A_n}$ and thus is in $S^\mu _L$ if $\sum _n \mu (A_n) \alpha _n \beta _n$ exists in *E*. For each $n\in \mathbb {N}$

76 $$\begin{aligned} 0 \le \mu (A_n) \alpha _n = \int j \mathbbm {1}_{A_n} {{\mathrm{\, \mathrm {d}}}}\mu \le \int h \mathbbm {1}_{A_n} {{\mathrm{\, \mathrm {d}}}}\mu = \nu (A_n), \end{aligned}$$

whence $0 \le \mu (A_n) \alpha _n b_n \le \nu (A_n) b_n$. Because $f\in S^{\nu +}_L$, $\sum _n \nu (A_n) b_n$ exists in *E*. Since *E* is splitting also $\sum _n \mu (A_n) \alpha _n b_n$ exists in *E*, i.e., $jf\in S^\mu _L$.

Furthermore, $\varphi ^\mu _L(jf) = \sum _n \mu (A_n) \alpha _n b_n \le \sum _n \nu (A_n) b_n = \varphi _L^\nu (f)$. On the other hand, we get $\mu (A_n)\alpha _n = \int j \mathbbm {1}_{A_n} {{\mathrm{\, \mathrm {d}}}}\mu \ge \frac{1}{s} \int h \mathbbm {1}_{A_n} {{\mathrm{\, \mathrm {d}}}}\mu = \frac{1}{s}\nu (A_n)$ for each $n\in \mathbb {N}$: it follows that $\varphi ^\mu _L( jf) \ge \frac{1}{s} \varphi _L^\nu (f)$. $\square$

### **Theorem 8.12**

Let *E* be Archimedean and splitting. Let $h: X \rightarrow [0,\infty )$ be measurable, $\nu := h \mu$.

- $$f \in S_{LV}^\nu \Longrightarrow hf \in S^\mu _{LV}, \varphi ^\mu _{LV}(hf) = \varphi ^\nu _{LV}(f)$$,
- $$f \in S_{VL}^\nu \Longrightarrow hf \in S_{VL}^\mu , \varphi _{VL}^\mu (hf) = \varphi _{VL}^\nu (f)$$.

### *Proof*

Since both $S^\nu _{LV}$ and $S^\nu _{VL}$ are directed, we assume $f \ge 0$.

- Let $f\in S_{LV}^{\nu +}$. For $n\in \mathbb {N}$ let $j_n$ be partially in $[\mathcal {A}]$ and such that $j_n \le h \le (1+ \frac{1}{n}) j_n$. Let $\Lambda , \Upsilon \subset S_L^\nu$ be countable sets with $\Lambda \le f \le \Upsilon$ be such that $\sup \varphi ^\nu _L(\Lambda ) = \varphi ^\nu _{LV}(f) = \inf \varphi ^\nu _L(\Upsilon )$. Then for all $\sigma \in \Lambda$ (note that $\sigma \in S_L^{\nu +}-S_L^{\nu +}$), $\tau \in \Upsilon$ and $n\in \mathbb {N}$ we have $j_n \sigma \le hf \le (1+ \frac{1}{n}) j_n \tau$ and by Lemma 8.11 $j_n \sigma$ and $(1+\frac{1}{n}) j_n \tau$ are in $S^\mu _L$. Therefore we are done if both $\inf _{n\in \mathbb {N},\sigma \in \Lambda , \tau \in \Upsilon } \varphi ^\mu _L((1+\frac{1}{n})j_n \tau - j_n \sigma )=0$ and $\varphi ^\mu _L(j_n \sigma )\le \varphi _{LV}^\nu (f) \le \varphi ^\mu _L((1+\frac{1}{n})j_n \tau )$ for all $n\in \mathbb {N}$ and all $\sigma \in \Lambda , \tau \in \Upsilon$. By Lemma 8.11 applied repeatedly we have
  77 $$\begin{aligned} 0&\le \varphi ^\mu _L((1+\tfrac{1}{n})j_n \tau - j_n \sigma ) = \varphi ^\mu _L(j_n \tau - j_n \sigma ) + \tfrac{1}{n} \varphi ^\mu _L(j_n \tau ) \nonumber \\&\le \varphi ^\nu _L(\tau - \sigma ) + \tfrac{1}{n} \varphi _L^\nu (\tau ), \end{aligned}$$
  which has infimum 0 since *E* is Archimedean and $\inf _{\tau \in \Upsilon ,\sigma \in \Lambda } \varphi _L^\nu (\tau - \sigma ) =0$. On the other hand, by Lemma 8.11,
  78 $$\begin{aligned} \varphi ^\mu (j_n \sigma )&\le \varphi ^\nu _L(\sigma ) \le \varphi ^\nu _{LV}(f) \le \varphi ^\nu _L(\tau ) \le (1+ \tfrac{1}{n}) \varphi ^\mu _L(j_n \tau ) \quad \nonumber \\&\quad \, (n\in \mathbb {N}, \sigma \in \Lambda , \tau \in \Upsilon ). \end{aligned}$$
- Let $f\in S_{VL}^{\nu +}$. Choose a partition $(A_n)_{n\in \mathbb {N}}$ with $f\mathbbm {1}_{A_n} \in S_V^\nu$ for $n\in \mathbb {N}$. By (a), $hf\mathbbm {1}_{A_n} \in S^\mu _{LV}$ for $n\in \mathbb {N}$; by Lemma 5.7 $h f\mathbbm {1}_{A_n}$ is partially in $S_V^\mu$. Therefore we can choose a partition $(B_n)_{n\in \mathbb {N}}$ with
  79 $$\begin{aligned} f\mathbbm {1}_{B_n} \in S_V^\nu , \quad h f\mathbbm {1}_{B_n} \in S_V^\mu \quad (n\in \mathbb {N}). \end{aligned}$$
  By (a), $\varphi _V^\nu ( f\mathbbm {1}_{B_n}) = \varphi _V^\mu (hf \mathbbm {1}_{B_n})$ for all $n\in \mathbb {N}$. But $f\in S^{\nu +}_{VL}$, so
  80 $$\begin{aligned} \varphi _{VL}^\nu (f) = \sum _n \varphi _V^\nu (f\mathbbm {1}_{B_n}) = \sum _n \varphi _V^\mu (hf \mathbbm {1}_{B_n}). \end{aligned}$$
  Then $$hf \in S_{VL}^\mu $$ and $$\varphi ^\mu _{VL}(hf) = \varphi ^\nu _{VL}(f)$$.$$\square $$

### **Theorem 8.13**

Let *E* be Archimedean and splitting. Let $h: X \rightarrow [0,\infty )$ be measurable, $\nu := h \mu$, $A= \{x\in X: h(x)>0\}$. Let $f: X \rightarrow E$ be such that $hf \in S^\mu _{LV}$. Then $f\mathbbm {1}_{A} \in S^\nu _{LV}$.

### *Proof*

Define $$h^*: X \rightarrow [0,\infty )$$ by

81 $$\begin{aligned} h^*(x) = {\left\{ \begin{array}{ll} \frac{1}{h(x)} &{} \text{ if } x\in A, \\ 0 &{} \text{ if } x\notin A. \end{array}\right. } \end{aligned}$$

Then $h^*$ is measurable and $hh^*= \mathbbm {1}_{A}$ and $\mathbbm {1}_A = \mathbbm {1}$ $\nu$-a.e.

*hf* is in $S^\mu _L$ and thus in $S_L^{\mathbbm {1}_A\mu }$, and since $\mathbbm {1}_A\mu = h^* \nu$, also $hf \in S_L^{h^*\nu }$. By Theorem 8.12, applied to $h^*, h^*\nu , \nu , hf$ instead of $h,\nu ,\mu ,f$, the function $h^*h f$ is an element of $S_{LV}^\nu$. But $h^* h f = \mathbbm {1}_A f$. $\square$

In Theorem 8.14 we show that extensions of simple functions with values in *E* composed with a $\sigma$-order continuous linear map $E\rightarrow F$ are extensions of simple functions with values in *F* (where *E* and *F* are Riesz spaces).

### **Theorem 8.14**

Let *E* and *F* be Riesz spaces. Let $S^E$ and $\varphi ^E$ be as in 4.33, and let $S^F$ and $\varphi ^F$ be defined analogously. Let $\mathcal {L}_c(E,F)$ denote the set of $\sigma$-order continuous linear functions $E\rightarrow F$ and $E_c^\sim = \mathcal {L}_c(E,\mathbb {R})$ (definition and notation as in Zaanen [13, Chapter 12,§84]). Let $f\in S^E_{LV}$. Then $\alpha \circ f \in S^F_{LV}$ for all $\alpha \in \mathcal {L}_c(E,F)$ and

82 $$\begin{aligned} \alpha \left( \varphi ^E_{LV}(f) \right) = \varphi ^F_{LV}( \alpha \circ f). \end{aligned}$$

In particular, $\alpha \circ f$ is integrable for all $\alpha \in E^\sim _c$, and $\alpha ( \varphi ^E_{LV}(f)) = \int \alpha \circ f {{\mathrm{\, \mathrm {d}}}}\mu$.

### *Proof*

Suppose $\alpha \in \mathcal {L}_c(E,F)^{+}$. Let $\tau \in S^{E+}_L$. Suppose $\tau = \sum _{n\in \mathbb {N}} a_n \mathbbm {1}_{A_n}$ for some partition $(A_n)_{n\in \mathbb {N}}$ and a sequence $(a_n)_{n\in \mathbb {N}}$ in $E^{+}$. Then $\alpha ( \varphi ^E_L( \tau )) = \alpha ( \sum _n \mu (A_n)a_n ) = \sum _n \mu (A_n) \alpha (a_n)$. Thus $\alpha \circ \tau$ is in $S^F_{LV}$ with $\alpha ( \varphi ^E_L( \tau )) = \varphi ^F_L (\alpha \circ \tau )$. Let $(\sigma _n)_{n\in \mathbb {N}}, (\tau _n)_{n\in \mathbb {N}}$ be sequences in $S^E_L$ with $\sigma _n \le f\le \tau _n$, $\sigma _n \uparrow , \tau _n \downarrow$ and $\varphi ^E_{LV}(f) = \sup _{n\in \mathbb {N}}\varphi ^E_L(\sigma _n)= \inf _{n\in \mathbb {N}}\varphi ^E_L(\tau _n)$. Then we have $\alpha ( \varphi ^E_{LV}(f)) = \sup _{n\in \mathbb {N}}\alpha (\varphi ^E_L(\sigma _n)) = \sup _{n\in \mathbb {N}}\varphi ^F_L( \alpha \circ \sigma _n )$ and $\alpha ( \varphi ^E_{LV}(f) ) = \inf _{n\in \mathbb {N}}\alpha (\varphi ^E_L(\tau _n)) =\inf _{n\in \mathbb {N}}\varphi ^F_L( \alpha \circ \tau _n )$. Since $\alpha \circ \sigma _n \le \alpha \circ f \le \alpha \circ \tau _n$ for all $n\in \mathbb {N}$, we conclude that $\alpha \circ f \in (S^F)_{LV}$ (see Theorem 7.1) with $\alpha ( \varphi ^E_{LV}(f))= \varphi ^F_{LV}(f)$. $\square$

Theorem 8.14 will be used in Sect. 9 to compare the integrals $\varphi _{LV}$ and $\varphi _{VL}$ with the Pettis integral.

Before proving Theorem 8.16 we state (in Theorem 8.15) that there is an equivalent formulation for a function *F* to be in $C(X\times T)$ whenever *X*, *T* are topological spaces and *X* is compact.

### **Theorem 8.15**

([14, Theorem 7.7.5]) Let *X* be a compact and let *T* be a topological space. Let $F: X\times T \rightarrow \mathbb {R}$ be such that $F(\cdot ,t) \in C(X)$ for all $t\in T$. Then $F\in C(X\times T)$ if and only if $t\mapsto F(\cdot ,t)$ is continuous, where *C*(*X*) is equipped with the supremum norm. Consequently, if $A\subset X$ is a compact set, then $t\mapsto \sup F(A,t)$ and $t\mapsto \inf F(A,t)$ are continuous.

### **Theorem 8.16**

Let $(X,d,\mu )$ be a compact metric probability space. Let *T* be a topological space and $F\in C(X \times T)$. The function $H: X \rightarrow C(T)$ given by $H(x)= F(x,\cdot )$ is an element of $S_V$. Furthermore, for $t\in T$, $x\mapsto F(x,t)$ is integrable and

83 $$\begin{aligned} \left[ \varphi _V (H) \right] (t) = \int F(x,t) {{\mathrm{\, \mathrm {d}}}}\mu (x) \quad (t\in T). \end{aligned}$$

### *Proof*

For $k\in \mathbb {N}$ let $A_{k1},\ldots , A_{kn_k}$ be a partition of *X* with ${{\mathrm{diam}}}A_{ki} \le k^{-1}$. Define

84 $$\begin{aligned} \Delta _k(t) = \sup _{x,y\in X, d(x,y)<k^{-1}} |F(x,t)- F(y,t)| \quad (t\in T). \end{aligned}$$

Since $x\mapsto F(x,t)$ is uniformly continuous for all $t\in T$, $\Delta _k(t) \downarrow 0$ for all $t\in T$. By Theorem 8.15 $t\mapsto \sup F(A_{ki},t)$ and $t\mapsto \inf F(A_{ki},t)$ are continuous for all $k\in \mathbb {N}$ and $i\in \{1,\ldots ,n_k\}$. For $k\in \mathbb {N}$ let $h_k, l_k : X \rightarrow C(T)$ be given by

85 $$\begin{aligned} h_k(x)&= \ t\mapsto \sup F(A_{ki},t) \quad (x\in A_{ki}),\nonumber \\ l_k(x)&= \ t\mapsto \inf F(A_{ki},t) \quad (x\in A_{ki}). \end{aligned}$$

Then $h_k,l_k\in S$ and $(h_k(x))(t) \ge F(x,t) \ge (l_k(x))(t)$ for all $x\in X$, $t\in T$. For $x\in A_{ki} \cap A_{mj}$ and $t\in T$

86 $$\begin{aligned} (h_k(x) - l_m(x) ) (t)&= \sup F( A_{ki},t) - \inf F(A_{mj},t) \nonumber \\&\le \sup \{ F(u,t) - F(v,t) : u,v \in A_{ki}\cup A_{mj} \} \le \Delta _{k\wedge m}(t). \end{aligned}$$

Let $a_k = \varphi (h_k )$ and $b_k = \varphi ( l_k)$ for $k\in \mathbb {N}$. Then $0 \le a_k(t) -b_m(t)\le \Delta _{k\wedge m}(t)$ for all $k,m\in \mathbb {N}$ and $\inf _{k,m\in \mathbb {N}} a_k(t) - b_m(t) \le \inf _{k\in \mathbb {N}}\Delta _k(t) =0$. Since $a_k,b_k\in C(T)$ and $\sup _{n\in \mathbb {N}}b_n (t) = \inf _{n\in \mathbb {N}}a_n(t)$ for all $t\in T$, the function $t \mapsto \inf _{n\in \mathbb {N}}a_n(t)$ is continuous, i.e., $x\mapsto F(x,\cdot )$ is an element of $S_V$. Furthermore, we conclude that the function $x\mapsto F(x,t)$ is integrable (by Theorem 7.1) and conclude (83). $\square$

### *Example 8.17*

Consider a metrisable locally compact group *G*. Let $X \subset G$ be a compact set and $\mu$ be a finite (positive) measure on $\mathcal {B}(X)$, the Borel-$\sigma$-algebra of *X*. Let $g\in C(G)$. Define the convolution of *g* and $\mu$ to be the function $g * \mu : G \rightarrow \mathbb {R}$ given by $g * \mu (t) = \int g(tx^{-1}) {{\mathrm{\, \mathrm {d}}}}\mu (x)$ for $t\in G$. For $x\in X$, let $L_x g \in C(G)$ be the function $t\mapsto g(tx^{-1})$. Then by Theorem 8.16, the function $f : X \rightarrow C(G)$ given by $f(x) = L_x g$ is in $S_V$ and $g * \mu = \varphi _V(f) \in C(G)$.

## Comparison with Bochner- and Pettis integral

**We consider the situation of Sect**. 8, **with an** $\mathbf{E}$ **that has the structure of a Banach lattice.** We write $\Vert \cdot \Vert$ for the norm on *E* and $E'$ for the dual of *E*. Then, next to our $\varphi _{LV}$ (and other extensions) there are the Bochner and the Pettis integrals. (We refer the reader to Hille and Phillips [3, Section 3.7] for background on both integrals.) We denote the set of Bochner (Pettis) integrable functions from the measure space $(X,\mathcal {A},\mu )$ into the Banach lattice *E* by $\mathfrak {B}$ ($\mathfrak {P}$) and the Bochner (Pettis) integral of an integrable function *f* by $\mathfrak {b}(f)$ ($\mathfrak {p}(f)$).

### **9.1**

By definition of the Bochner integral, where one also starts with defining the integral on simple functions: $S\subset \mathfrak {B}$ and $\varphi = \mathfrak {b}$ on *S*. Since $\mathfrak {B}\subset \mathfrak {P}$ and $\mathfrak {b}= \mathfrak {p}$ on $\mathfrak {B}$ we also have $S\subset \mathfrak {P}$ with $\varphi = \mathfrak {p}$ on *S*.

### **9.2**

The following is used in this section. The Banach dual of *E* is equal to the order dual, i.e., $E' = E^\sim$. Moreover, for $x,y\in E$ (see de Jonge and van Rooij [15, Theorem 10.2])

87 $$\begin{aligned} x\le y \quad \iff \quad \alpha ( x) \le \alpha (y) \text{ for } \text{ all } \alpha \in E^{\sim +}. \end{aligned}$$

This implies that for a sequence $(y_n)_{n\in \mathbb {N}}$ and *x*, *y* in *E*:

88 $$\begin{aligned} \inf _{n\in \mathbb {N}}\alpha (y_n)=0 \text{ for } \text{ all } \alpha \in E^{\sim +} \quad&\Longrightarrow \quad \inf _{n\in \mathbb {N}}y_n =0. \end{aligned}$$

### **Theorem 9.3**

Let $f\in \mathfrak {P}^{+}$ and *f* be partially in *S*. Then $f\in S_L^{+}$ and $\mathfrak {p}(f) = \varphi _L(f)$.

### *Proof*

Let $(A_n)_{n\in \mathbb {N}}$ be a partition for which $f_n := f\mathbbm {1}_{A_n} \in S$. Then for every $\alpha \in E^{\sim +}$

89 $$\begin{aligned} \alpha ( \mathfrak {p}(f) ) = \int \alpha \circ f {{\mathrm{\, \mathrm {d}}}}\mu = \sum _{n\in \mathbb {N}}\int \alpha \circ f_n {{\mathrm{\, \mathrm {d}}}}\mu = \sum _{n\in \mathbb {N}}\alpha ( \varphi (f_n)). \end{aligned}$$

Hence $\inf _{N\in \mathbb {N}}\alpha ( \mathfrak {p}(f) - \sum _{n=1}^N \varphi (f_n)) =0$ and thus $\mathfrak {p}(f) = \sum _n \varphi (f_n)$ (see (88)). $\square$

### **Theorem 9.4**

Let $f\in \mathfrak {P}$. Then the following holds.

- If $g\in S_{LV}$ and $f\le g$, then $\mathfrak {p}(f) \le \varphi _{LV}(g)$.
- If $S_V$ is stable, $g\in S_{VLV}$ and $f\le g$, then $\mathfrak {p}(f) \le \varphi _{VLV}(g)$.

Consequently, $\mathfrak {p}= \varphi _{LV}$ on $\mathfrak {P}\cap S_{LV}$, and $\mathfrak {p}= \varphi _{VLV}$ on $\mathfrak {P}\cap S_{VLV}$ if $S_V$ is stable.

The statements in (a) and (b) remain valid by replacing all “$\le$” by “$\ge$”.

### *Proof*

It will be clear that if $g\in S$ and $f\le g$, then $g\in \mathfrak {P}$ and hence $\mathfrak {p}(f) \le \mathfrak {p}(g) =\varphi (g)$.

If $g\in S_V$ and $f\le g$, then there exists an $\Upsilon \subset S$ with $g \le \Upsilon$ and $\varphi _V(g) = \inf \varphi (\Upsilon ) = \inf \mathfrak {p}(\Upsilon ) \ge \mathfrak {p}(f)$.

Let $g\in S_L$ and assume $f\le g$. Let $g_1,g_2\in S_L^{+}$ be such that $g= g_1 - g_2$. Let $(B_i)_{i\in \mathbb {N}}$ be a $\varphi$-partition for both $g_1$ and $g_2$. Write $A_n = \bigcup _{i=1}^n B_i$ for $n\in \mathbb {N}$. Let $\alpha \in E^{\sim +}$. $\alpha \circ (f\mathbbm {1}_A) = (\alpha \circ f) \mathbbm {1}_A$ for every $A\in \mathcal {A}$, so that $\alpha \circ (f\mathbbm {1}_A)$ is integrable. Thus, for $n\in \mathbb {N}$ we have

90 $$\begin{aligned} \int (\alpha \circ f)\mathbbm {1}_{A_n} {{\mathrm{\, \mathrm {d}}}}\mu&= \int \alpha \circ (f\mathbbm {1}_{A_n}) {{\mathrm{\, \mathrm {d}}}}\mu \le \int \alpha \circ (g \mathbbm {1}_{A_n}) {{\mathrm{\, \mathrm {d}}}}\mu \nonumber \\&= \int \alpha \circ g_1 \mathbbm {1}_{A_n} {{\mathrm{\, \mathrm {d}}}}\mu - \int \alpha \circ g_2 \mathbbm {1}_{A_n} {{\mathrm{\, \mathrm {d}}}}\mu \nonumber \\&= \alpha (\varphi (g_1 \mathbbm {1}_{A_n})) - \alpha (\varphi (g_2 \mathbbm {1}_{A_n}))\nonumber \\&\le \alpha (\varphi (g_1 \mathbbm {1}_{A_m})) - \alpha (\varphi (g_2 \mathbbm {1}_{A_k})) \quad (k,m\in \mathbb {N}, k<n<m). \end{aligned}$$

Which implies that $\int (\alpha \circ f)\mathbbm {1}_{A_n} {{\mathrm{\, \mathrm {d}}}}\mu + \alpha (\varphi (g_2 \mathbbm {1}_{A_k})) \le \alpha ( \varphi _L(g_1))$ as soon as $k<n$. By letting *n* tend to $\infty$ (as $\int (\alpha \circ f)\mathbbm {1}_{A_n} {{\mathrm{\, \mathrm {d}}}}\mu \rightarrow \int \alpha \circ f {{\mathrm{\, \mathrm {d}}}}\mu = \alpha (\mathfrak {p}(f))$), for each $k\in \mathbb {N}$ we obtain

91 $$\begin{aligned} \alpha (\mathfrak {p}(f)) \le \alpha (\varphi _L(g_1) - \varphi (g_2 \mathbbm {1}_{A_k})). \end{aligned}$$

This holds for all $\alpha \in E^{\sim +}$, so

92 $$\begin{aligned} \mathfrak {p}(f) \le \varphi _L(g_1) - \varphi (g_2 \mathbbm {1}_{A_k}) . \end{aligned}$$

This, in term is true for every *k*, so $\mathfrak {p}(f)) \le \varphi _L(g)$.

We leave it to check that the preceding lines can be repeated with $S_V$, $S_L$ or $S_{VL}$ instead of *S*. $\square$

### **Theorem 9.5**

Suppose $\Vert \cdot \Vert$ is $\sigma$-order continuous. Write $\overline{S} = S_{LV} = S_{VL}$ and $\overline{\varphi }= \varphi _{LV} = \varphi _{VL}$ (see Theorem 5.12).

- Then $\overline{S} \subset \mathfrak {P}$. Consequently, if *f* is essentially separably valued and in $\overline{S}$, then $f\in \mathfrak {B}$. In particular, $S_L \subset \mathfrak {B}$.
- Suppose there exists an $\alpha \in E^{\sim +}_c$ with the property that if $b\in E$ and $b>0$, then $\alpha (b)>0$. Then $\mathfrak {B}_V \subset \mathfrak {B}$. Consequently, $\overline{S} \subset \mathfrak {B}$.

### *Proof*

- Because $\Vert \cdot \Vert$ is $\sigma$-order continuous, $E'= E^\sim _c$. Therefore Theorem 8.14 implies that $\overline{S} \subset \mathfrak {P}$. Note that $S_L \subset \mathfrak {B}$. Since $\mathfrak {B}$ is a Riesz ideal in the space of strongly measurable functions $X\rightarrow E$, an $f\in \overline{S}$ is an element of $\mathfrak {B}$ if it is essentially separably valued, since there are elements $\sigma ,\tau \in S_L$ with $\sigma \le f \le \tau$ and *f* is weakly measurable since $f\in \mathfrak {P}$.
- Suppose $f\in \mathfrak {B}_V$ and $\sigma _n, \tau _n \in \mathfrak {B}$ are such that $\sigma _n \le f \le \tau _n$ for $n\in \mathbb {N}$, $\sigma _n \uparrow , \tau _n \downarrow$ and $\sup _{n\in \mathbb {N}}\mathfrak {b}(\sigma _n) = \mathfrak {b}_V(f) = \inf _{n\in \mathbb {N}}\mathfrak {b}(\tau _n)$. Then $\inf _{n\in \mathbb {N}}\int \alpha \circ (\tau _n - \sigma _n) {{\mathrm{\, \mathrm {d}}}}\mu = \alpha ( \inf _{n\in \mathbb {N}}\mathfrak {b}( \tau _n - \sigma _n ) ) = 0$ and therefore $\alpha ( \inf _{n\in \mathbb {N}}(\tau _n - \sigma _n)) = \inf _{n\in \mathbb {N}}\alpha \circ (\tau _n - \sigma _n)$ is integrable with integral equal to zero. Therefore $\inf _{n\in \mathbb {N}}(\tau _n - \sigma _n)=0$ a.e., hence $\tau _n \rightarrow f$ a.e. Therefore *f* is strongly measurable and thus $f\in \mathfrak {B}$ by (a). By (a) $S_L \subset \mathfrak {B}$, hence $\overline{S} = S_{LV} \subset \mathfrak {B}$.

$$\square $$

### **9.6**

For the next theorem we write $S_\mathbb {R}$ for the space of simple functions $X\rightarrow \mathbb {R}$. Note that if $u\in E^{+}$ and $\pi$ is an element of $(S_{\mathbb {R}})^{+}_L$, thus of $\mathcal {L}^1(\mu )$, then $u\pi \in S_L$ and $\varphi _L(u\pi ) = u \int \pi {{\mathrm{\, \mathrm {d}}}}\mu$.

### **Theorem 9.7**

Let *Y* be a compact Hausdorff space and let $E=C(Y)$ be equipped with the supremum norm, $\Vert \cdot \Vert _\infty$. Then $\mathfrak {B}\subset S_{LV}$ and $\varphi _{LV}= \mathfrak {b}$ on $\mathfrak {B}$.

### *Proof*

Let $f\in \mathfrak {B}$ and $(s_n)_{n\in \mathbb {N}}$ be a sequence of simple functions $X\rightarrow E$ such that $\int \Vert f-s_n \Vert _\infty {{\mathrm{\, \mathrm {d}}}}\mu \le \frac{1}{n 2^n}$ for all *n*. Then there exists an integrable function $g: X \rightarrow [0,\infty )$ with $g \ge \sum _{n\in \mathbb {N}} n \Vert f-s_n \Vert _\infty$ $\mu$-a.e., and thus $g\ge n \Vert f - s_n\Vert _\infty$ $\mu$-a.e. By Theorem 7.1 there exists a $\pi \in (S_\mathbb {R})_L^{+}$ (see 9.6) with $\pi \ge g$. Then, let $Z\in \mathcal {A}$ with $\mu (A) =0$ be such that $\Vert f(x)- s_n(x)\Vert _\infty \le \frac{1}{n} \pi (x)$ for all $x\in X {\setminus } Z$. We may, by replacing $s_n$ by $s_n \mathbbm {1}_{X{\setminus } Z}$, assume that $s_n =0$ on *Z*. With $Z_k = \{ x\in Z: k-1<\Vert f(x)\Vert _\infty \le k\}$ and $\rho$ the element in $S_L^{+}$ with $\rho (x) = 0$ for $x\notin Z$ and $\rho (x) = k \mathbbm {1}_Y$ for $x\in Z_k$, we have

93 $$\begin{aligned}&s_n - \tfrac{1}{n} \mathbbm {1}_Y \pi - \rho \le f \le s_n + \tfrac{1}{n} \mathbbm {1}_Y \pi + \rho \quad (n\in \mathbb {N}). \end{aligned}$$

Because $\varphi _L(\rho )=0$, $\varphi _L( \tfrac{1}{n} \mathbbm {1}_Y \pi ) \rightarrow 0$, $\varphi _L(s_n) = \mathfrak {b}(s_n)$ and $\mathfrak {b}(s_n) \rightarrow \mathfrak {b}(f)$, both $\varphi _L(s_n - \tfrac{1}{n} \mathbbm {1}_Y \pi - \rho )$ and $\varphi _L(s_n + \tfrac{1}{n} \mathbbm {1}_Y \pi + \rho )$ converge to $\mathfrak {b}(f)$. Whence

94 $$\begin{aligned}&\mathfrak {b}(f) = \sup _{n\in \mathbb {N}}\varphi _L( s_n - \tfrac{1}{n} \mathbbm {1}_Y\pi -\rho ) = \inf _{n\in \mathbb {N}}\varphi _L( s_n + \tfrac{1}{n} \mathbbm {1}_Y \pi +\rho ) . \end{aligned}$$

Thus $$f\in S_{LV}$$ and $$\varphi _{LV}(f) = \mathfrak {b}(f)$$. $$\square $$

By the Yosida Representation Theorem the following is an immediate consequence.

### **Corollary 9.8**

Let *E* be a Archimedean Riesz space with strong unit *u* and assume *E* is uniformly complete, i.e., *E* is a Banach lattice under the norm $\Vert \cdot \Vert _u$ given by $\Vert x\Vert _u = \inf \{ \lambda \in [0,\infty ) : |x|\le \lambda u\}$. Then $\mathfrak {B}\subset S_{LV}$ and $\varphi _{LV}= \mathfrak {b}$ on $\mathfrak {B}$.

### *Example 9.9*

(I) Take $X=\mathbb {N}$, $\mathcal {A}= \mathcal {P}(\mathbb {N})$, and let $\mu$ be the counting measure. We have $S= c_{00}[E]$; $S_V=c_{00}[E]$; all functions $\mathbb {N}\rightarrow E$ are partially in *S*; $\overline{S}:= S_{LV} = S_{VL}=S_L$ (see Theorem 5.5(c)) and $\overline{S}^{+}$ consists precisely of the functions $f: \mathbb {N}\rightarrow E^{+}$ for which $\sum _n f(n)$ exists in the sense of the ordering. On the other hand, $f: \mathbb {N}\rightarrow E$ is Bochner integrable if and only if $\sum _{n=1}^\infty \Vert f(n)\Vert <\infty$.
  If $\Vert \cdot \Vert$ is a $\sigma$-order continuous norm, then $\mathfrak {B}\subset \overline{S}$.
  Moreover $\Vert \cdot \Vert$ is equivalent to an abstract L-norm if and only if $\mathfrak {B}= \overline{S}$ (since, if $\mathfrak {B}=\overline{S}$, the following holds: if $x_1,x_2,\ldots \in E^{+}$ and $\sum _n x_n$ exists, then $\sum _{n\in \mathbb {N}}\Vert x_n\Vert <\infty$, see Theorem 12.1).
  For $E=c_0$ there exists an $f\in \mathfrak {P}$ that is not in $\overline{S}$. For example $f: \mathbb {N}\rightarrow c_0$ given by
    95 $$\begin{aligned} f= (e_1,-e_1,e_2,-e_2,e_3,-e_3,\ldots ) \end{aligned}$$
    is Pettis integrable since $c_0'\cong \ell ^1$ has basis $\{\delta _n:n\in \mathbb {N}\}$ where $\delta _n(x) = x(n)$ and $\sum _{m\in \mathbb {N}} \delta _n(f(m)) =0$ for all $m\in \mathbb {N}$. $c_0$ is $\sigma$-Dedekind complete and thus by Theorem 4.32 the set $\overline{S}$ is a Riesz space. However, |*f*| is not in $\overline{S}$ and therefore neither *f* is.
  For $E=c$ there exists an $f\in \overline{S}$ that is not in $\mathfrak {B}$ and not in $\mathfrak {P}$: Consider for example $f: n \mapsto e_n$. It is an element of $\overline{S}$ but not of $\mathfrak {B}$. It is not even Pettis integrable. (Suppose it is, and its integral is *a*. Then for all $u\in c'$ we have $u(a)= \int u \circ f {{\mathrm{\, \mathrm {d}}}}\mu = \sum _{n=1}^\infty u(f(n)) = \sum _{n=1}^\infty u(e_n)$. Letting *u* be the coordinate functions, we see that $a(n)=1$ for all $n\in \mathbb {N}$; letting *u* be $x\mapsto \lim _{n\rightarrow \infty }x(n)$ we have a contradiction.)
(II) $\mathfrak {B}\not \subset S_{VLV}$. Let $(\mathbb {R}, \mathcal {M}, \lambda )$ be the Lebesgue measure space. Let *E* be the $\sigma$-Dedekind complete Riesz space $L^1(\lambda )$. Let $g\in L^1(\lambda )$ be the equivalence class of the function that equals $t^{-\frac{1}{2}}$ for $0<t\le 1$ and equals 0 for other *t*. Let $L_x g(t) = g(t-x)$ for $x\in \mathbb {R}$. Then the function $f: \mathbb {R}\rightarrow L^1(\lambda )$ for which $f(x)=\mathbbm {1}_{[0,1]}(x) L_x g$ is Bochner integrable (*f* is continuous in the $\Vert \cdot \Vert _1$ norm (because $\Vert L_\varepsilon g - g\Vert _1 = 2\sqrt{\varepsilon }$ for $\varepsilon >0$) and $\int \Vert f(x)\Vert _1 {{\mathrm{\, \mathrm {d}}}}\lambda (x) = \int \int |g(t-x)| {{\mathrm{\, \mathrm {d}}}}\lambda (t) {{\mathrm{\, \mathrm {d}}}}\lambda (x) =\Vert g\Vert _1 <\infty$) but no element of $S_{VLV}$ (by Theorem 8.3).

## Extensions of Bochner integrable functions

**Consider the situation of Sect**. 9.

As we have seen in Examples 9.9, e.g., (95), the set of Pettis integrable functions need not be stable. We show that $\mathfrak {B}$ is stable and $\mathfrak {b}$ is laterally extendable. Furthermore we give an example of an $f\in \mathfrak {B}_{LV}$ that is neither in $S_{VLV}$, nor in $\mathfrak {B}_L$ or $\mathfrak {B}_V$.

### **Theorem 10.1**

$\mathfrak {B}$ is stable and $\mathfrak {b}$ is laterally extendable.

### *Proof*

Note that $f\mathbbm {1}_B \in \mathfrak {B}$ for all $f\in \mathfrak {B}$ and $B\in \mathcal {A}$ (since $f\mathbbm {1}_B$ is strongly measurable and $\Vert f\mathbbm {1}_B\Vert$ is integrable), i.e., $\mathfrak {B}$ is stable. Let $(A_n)_{n\in \mathbb {N}}$ be a partition in $\mathcal {A}$ of *X*. Let $f: X \rightarrow E^{+}$ be a Bochner integrable function. Then $\int \Vert f\Vert {{\mathrm{\, \mathrm {d}}}}\mu <\infty$ and with $B_n = A_1 \cup \cdots \cup A_n$ and Lebesgue’s Dominated Convergence Theorem we obtain

96 $$\begin{aligned} \left\| \mathfrak {b}\left( f - \sum _{n=1}^N f \mathbbm {1}_{A_n}\right) \right\| \le \int \Vert f(x) - \mathbbm {1}_{B_N}(x) f(x) \Vert {{\mathrm{\, \mathrm {d}}}}\mu (x) \rightarrow 0. \end{aligned}$$

Thus

97 $$\begin{aligned} \mathfrak {b}( f ) = \lim _{N\rightarrow \infty }\sum _{n=1}^N \mathfrak {b}( f \mathbbm {1}_{A_n} ) = \sum _{n} \mathfrak {b}( f \mathbbm {1}_{A_n}). \end{aligned}$$

We conclude that $\mathfrak {b}$ is laterally extendable. $\square$

In the following situation we have $\mathfrak {B}_L= \mathfrak {B}= \mathfrak {B}_V$.

### **Lemma 10.2**

Let *E* be a Banach lattice with an abstract L-norm (i.e., $\Vert a+b\Vert =\Vert a\Vert +\Vert b\Vert$ for $a,b\in E^{+})$.

- Then
  98 $$\begin{aligned} \Vert \mathfrak {b}(f)\Vert = \int \Vert f\Vert {{\mathrm{\, \mathrm {d}}}}\mu \quad (f\in \mathfrak {B}^{+}). \end{aligned}$$
- $$\mathfrak {B}_L= \mathfrak {B}$$.
- There exist an $\alpha \in E^{\sim +}_c$ as in Theorem 9.5(b). Consequently $\mathfrak {B}_V = \mathfrak {B}$.

### *Proof*

- It is clear that $\Vert \mathfrak {b}(f)\Vert = \int \Vert f\Vert {{\mathrm{\, \mathrm {d}}}}\mu$ for $f\in S^{+}$, hence by limits for all $f\in \mathfrak {B}^{+}$.
- Suppose $f\in \mathfrak {B}_L^{+}$. Let $(A_n)_{n\in \mathbb {N}}$ be a $\mathfrak {b}$-partition for *f*, write $f_n = f\mathbbm {1}_{A_n}$. Then $\Vert \sum _{n=1}^N f_n - f\Vert \rightarrow 0$, hence *f* is strongly measurable. Moreover, since $\Vert \cdot \Vert$ is $\sigma$-order continuous $\Vert \sum _{n=1}^N \mathfrak {b}(f_n) - \mathfrak {b}_L(f)\Vert \rightarrow 0$, hence $\sum _{n=1}^N \Vert \mathfrak {b}(f_n) \Vert \rightarrow \mathfrak {b}_L(f)$. Using (a) we obtain $\int \Vert f\Vert {{\mathrm{\, \mathrm {d}}}}\mu = \sum _{n\in \mathbb {N}}\int \Vert f_n\Vert {{\mathrm{\, \mathrm {d}}}}\mu = \sum _{n\in \mathbb {N}}\Vert \mathfrak {b}(f_n)\Vert <\infty$, i.e., $f\in \mathfrak {B}$.
- Extend $\alpha : E^{+} \rightarrow \mathbb {R}$ given by $\alpha (b)=\Vert b\Vert$ to a linear map on *E*.

$$\square $$

### **10.3**

Consider the situation of Example 8.8. Since $S\subset \mathfrak {B}$ and $\varphi (h) = \mathfrak {b}( h )$ for $h\in S$: $f\in \mathfrak {B}_V$. The function *f* is not essentially separably-valued (i.e., $f(X{\setminus } A)$ is not separable for all null sets $A\in \mathcal {A})$, hence *f* (and thus *g*) is not strongly measurable (see [3, Theorem3.5.2]). Hence *f* is not Bochner integrable, i.e., $f\in \mathfrak {B}_V$ but $f\notin \mathfrak {B}$.

In a similar way as has been shown in Example 8.8, one can show that $g: \mathbb {R}\rightarrow E^{+}$ defined by $g(t) = \mathbbm {1}_{\{t\}}$ for $t\in \mathbb {R}$ is in $S_{LV}$. Then $g \in \mathfrak {B}_{LV}$ but $g \notin \mathfrak {B}_{V}$.

### **10.4**

All $f\in \mathfrak {B}_L$ are strongly measurable. Therefore for $f\in \mathfrak {B}_L$ we have $f\notin \mathfrak {B}$ if and only if $\int \Vert f\Vert {{\mathrm{\, \mathrm {d}}}}\mu = \infty$.

The following example illustrates that by extending the Bochner integrable functions one can obtain more than by extending the simple functions.

### *Example 10.5*

[$$\psi \in \mathfrak {B}_{V}, \psi \notin \mathfrak {B}$$]

Let $X=[2,3]$, let $\mathcal {A}$ be the set of Lebesgue measurable subsets of *X* and $\mu$ be the Lebesgue measure on *X*. Let *M* denote the set of equivalence classes of measurable functions $\mathbb {R}\rightarrow \mathbb {R}$. Let

99 $$\begin{aligned} E= \Big \{ f \in M: \sup _{x\in \mathbb {R}} \int _{x}^{x+1} |f| <\infty \Big \}, \quad \Vert \cdot \Vert : E \rightarrow [0,\infty ), \quad \Vert f\Vert = \sup _{x\in \mathbb {R}} \int _{x}^{x+1} |f|. \end{aligned}$$

Then *E* equipped with the norm $\Vert \cdot \Vert$ is a Banach lattice. *E* is an ideal in *M* and therefore $\sigma$-Dedekind complete (hence $S_V$ is stable; 4.25). The norm $\Vert \cdot \Vert$ is not $\sigma$-order continuous.

For $a\in \mathbb {R}$, $c > 0$ define $S_{a,c}: X \rightarrow E^{+}$ by $S_{a,c}(x) = \mathbbm {1}_{(a+cx,\infty )}$. If $x,y\in X$ with $y>x$ then $\Vert S_{a,c}(x) - S_{a,c}(y)\Vert \le \Vert \mathbbm {1}_{(a+cx,a+cy]}\Vert \le c|x-y|$, so $S_{a,c}$ is continuous and therefore strongly measurable. Furthermore $\Vert S_{a,c}(x)\Vert = 1$ for all $x\in X$, i.e., $x\mapsto \Vert S_{a,c}(x)\Vert$ is integrable. Thus $S_{a,c}$ is Bochner integrable. For $d,e\in \mathbb {R}$ with $e>d$ the map $E \rightarrow \mathbb {R}$, $f \mapsto \int _d^e f$ is a continuous linear functional. Therefore

100 $$\begin{aligned} \int _{d}^e&\mathfrak {b}(S_{a,c}) = \int _X \int _{d}^e (S_{a,c}(x))(t) {{\mathrm{\, \mathrm {d}}}} t {{\mathrm{\, \mathrm {d}}}} x = \int _{d}^e \int _X (S_{a,c}(x))(t) {{\mathrm{\, \mathrm {d}}}} x {{\mathrm{\, \mathrm {d}}}} t. \end{aligned}$$

Since this holds for all $d,e\in \mathbb {R}$ with $e>d$, for $t\in \mathbb {R}$ we have

101 $$\begin{aligned} (\mathfrak {b}(S_{a,c}))(t) = \int _X (S_{a,c}(x))(t) {{\mathrm{\, \mathrm {d}}}} x = \int _2^3 \mathbbm {1}_{(a+cx,\infty )}(t) {{\mathrm{\, \mathrm {d}}}} x = \left( \tfrac{t-a}{c} \wedge 3 -2 \right) \vee 0. \end{aligned}$$

For $k\in \mathbb {N}$ define $r_k, R_k : X \rightarrow E$ by

102 $$\begin{aligned} R_k&:= S_{0,k}, \quad r_k:= S_{0,k}- S_{1,k}. \end{aligned}$$

For $x\in X$ and $k\in \mathbb {N}$, $r_k(x) = \mathbbm {1}_{(kx,kx+1]}$ and $kx+1 <(k+1)x$. Define

103 $$\begin{aligned} \psi (x) := \mathbbm {1}_{\bigcup _{k\in \mathbb {N}} (kx, kx+1]} = \sum _{k\in \mathbb {N}} r_k(x), \quad \sigma _n&:= \sum _{k=1}^n r_k , \quad \tau _n := \sum _{k=1}^n r_k + R_{n+1}. \end{aligned}$$

Note that $\sigma _n \le \psi \le \tau _n$ and $\sigma _n, \tau _n \in \mathfrak {B}$ all for $n\in \mathbb {N}$. Since *E* is $\sigma$-Dedekind complete and therefore mediated, from the fact that

104 $$\begin{aligned} \inf _{n\in \mathbb {N}}\mathfrak {b}( \tau _n - \sigma _n) = \inf _{n\in \mathbb {N}}\mathfrak {b}( R_{n+1}) = 0, \end{aligned}$$

it follows that $\psi \in \mathfrak {B}_V$. However, $\psi \notin \mathfrak {B}$ since $\psi$ is not essentially separably valued:

Let $x,y\in X$, $x<y$. We prove $\Vert \psi (x) - \psi (y) \Vert \ge 1$. For $k \in \mathbb {N}$:

105 $$\begin{aligned} k-1 \le \tfrac{1}{y-x} < k&\Longrightarrow {\left\{ \begin{array}{ll} 1+(k-1)y \le k x, \\ 1+kx < ky, \end{array}\right. } \nonumber \\&\Longrightarrow (kx, kx +1] \cap \bigcup _{i\in \mathbb {N}} (i y,i y+1] =\emptyset . \end{aligned}$$

Hence $\Vert \psi (x)- \psi (y)\Vert \ge 1$ for all $x,y\in X$ with $x \ne y$.

So $\psi$ is an element of $\mathfrak {B}_V$ but not of $\mathfrak {B}$ (and neither of $\mathfrak {B}_L$).

### *Example 10.6*

[$$f\in \mathfrak {B}_{LV}, f\notin \mathfrak {B}_L, f\notin \mathfrak {B}_V, f\notin S_{VLV}$$]

Let $(X,\mathcal {A},\mu )$ be the Lebesgue measure space $(\mathbb {R},\mathcal {M},\lambda )$. Let *E* and $\psi$ be as in Example 10.5. Define $u: \mathbb {R}\rightarrow E$ by

106 $$\begin{aligned} u(x) = {\left\{ \begin{array}{ll} \psi (x) &{} x\in [2,3], \\ 0 &{} \text {otherwise}. \end{array}\right. } \end{aligned}$$

Then *u* is an element of $\mathfrak {B}_V$ and not of $\mathfrak {B}_L$. As we have seen in Examples 9.9(II) there exists a *g* in $L^1(\lambda )$ and thus in *E* such that $v : x\mapsto \mathbbm {1}_{[0,1]}(x) L_x g$ is an element of $\mathfrak {B}$ that is not an element of $S_{VLV}$. Furthermore $w: \mathbb {R}\rightarrow E$ given by $w(x) = \mathbbm {1}_{(n,n+1]}$ for $x\in (n,n+1]$ is an element of $\mathfrak {B}_L$ and not of $\mathfrak {B}_V$. Therefore $f=u+v+w$ is an element of $\mathfrak {B}_{LV}$ (and thus of $\mathfrak {B}_{VL}$; see Theorem 5.8) but is neither an element of $S_{VLV}$ nor of $\mathfrak {B}_V$ or $\mathfrak {B}_L$.

## Discussion

Of course, to some extent our approach is arbitrary. We mention some alternatives, with comments.

### **11.1**

The reader may have wondered why in our definition of the lateral extension the sets $A_n$ are required not only to be disjoint but also to cover *X* (i.e., to form a partition). Without the covering of *X* the definition remains perfectly meaningful, but the sum of two positive laterally integrable functions need not be laterally integrable, even in quite natural situations. (E.g., take $E=F=\mathbb {R}$ and $X=[0,1]$; let $\mathcal {I}$ be the ring generated by the open intervals, $\Gamma$ the space of all Riemann integrable functions on [0, 1], and $\varphi$ the Riemann integral. If *f* is the indicator of the Cantor set, then $\mathbbm {1}-f$ is laterally integrable but $2\mathbbm {1} - f$ is not.)

### **11.2**

For the vertical extension we have, somewhat artificially, introduced a countability restriction leading us from $\varphi _v$ to $\varphi _V$; see Definition 3.3. In some sense, $\varphi _v$ would have served as well as $\varphi _V$. In order to get a non-void theory, however, we would need a much stronger (but analogous) condition than “mediatedness”, restricting our world drastically.

### **11.3**

A different approach to both the vertical and the lateral extension, closer to Daniell and Bourbaki, could run as follows. Starting from the situation of 3.14, call a function $X \rightarrow F^{+}$ “integrable” if there exist $f_1,f_2,\ldots \in \Gamma ^{+}$ such that

107 $$\begin{aligned} {\left\{ \begin{array}{ll} f_n \uparrow f \text{ in } F^X, \\ \sup _{n\in \mathbb {N}}\varphi (f_n) \text{ exists } \text{ in } E, \end{array}\right. } \end{aligned}$$

then define the “integral” $\overline{\varphi }(f)$ of *f* by

108 $$\begin{aligned} \overline{\varphi }(f) : = \sup _{n\in \mathbb {N}}\varphi (f_n) . \end{aligned}$$

This definition is meaningful only if, in the above situation

109 $$\begin{aligned} g\in \Gamma ^{+}, g \le f, \quad \Longrightarrow \quad \varphi (g) \le \sup _{n\in \mathbb {N}}\varphi (f_n) \end{aligned}$$

which in a natural way leads to the requirement that $\Gamma$ be a lattice and that $\varphi$ be continuous in the following sense:

110 $$\begin{aligned} h_1, h_2,\ldots \in \Gamma ^{+}, h_n \downarrow 0 \quad \Longrightarrow \quad \varphi (h_n) \downarrow 0. \end{aligned}$$

These conditions lead to a sensible theory, but again we consider them as too restrictive. (See Example II.2.4 in the thesis of Jeurnink [16] for an example of a $\Gamma$ that consists of simple functions on a measure space with values in a *C*(*X*) for which (110) does not hold for the standard integral on simple functions (see 4.33).)

## Appendix

### **Theorem 12.1**

Let *E* be a Banach lattice with the property

12.1 $$\begin{aligned} \text{ If } x_1,x_2,\ldots \in E^{+} \text{ and } \sum _n x_n \text{ exists, } \text{ then } \sum _{n\in \mathbb {N}} \Vert x_n\Vert <\infty . \end{aligned}$$

Then the norm $\Vert \cdot \Vert$ is equivalent to an L-norm.

The proof uses the following lemma.

### **Lemma 12.2**

Let *E* be a Banach lattice that satisfies (12.1). Then there exists a $C>0$ such that

2 $$\begin{aligned} x_1,x_2,\ldots \in E^{+}, \sum _n x_n \text{ exists } \quad \Longrightarrow \quad \sum _{n\in \mathbb {N}} \Vert x_n\Vert \le C \Big \Vert \sum _n x_n \Big \Vert . \end{aligned}$$

### *Proof*

Suppose not. For $i\in \mathbb {N}$ let $x_{i1}, x_{i2},\ldots \in E^{+}$, $\sum _n x_{in} = b_i$ and $\sum _{n\in \mathbb {N}} \Vert x_{in}\Vert > 2^i \Vert b_i\Vert$ and $\Vert b_i\Vert = 2^{-i}$. Then $\sum _{i\in \mathbb {N}} \Vert b_i\Vert <\infty$, so $\sum _i b_i$ exists. As $\sum _i b_i = \sum _i \sum _n x_{in}$, by (12.1) we get $\infty > \sum _{i\in \mathbb {N}} \sum _{n\in \mathbb {N}} \Vert x_{in}\Vert > \sum _{i\in \mathbb {N}} 2^i \Vert b_i\Vert = \infty$. $\square$

### *Proof of Theorem 12.1*

By Lemma 12.2 we can define $p: E \rightarrow [0,\infty )$,

3 $$\begin{aligned} p(x)= \sup \left\{ \sum _{n\in \mathbb {N}} \Vert x_n\Vert \ : \ x_1,x_2,\ldots \in E^{+}, \sum _n x_n \le |x| \right\} , \end{aligned}$$

obtaining $p(x)=p(|x|), p(tx) = |t|p(x), \Vert x\Vert \le p(x)\le C \Vert x\Vert$ for all $x\in E$, $t\in \mathbb {R}$ (with *C* as in Lemma 12.2) and $p(x) \le p(y)$ for $x,y\in E^{+}$ with $x\le y$.

Let $x,y\in E^{+}$; we prove $p(x +y) = p(x)+p(y)$.

- For $\varepsilon >0$ choose $x_1,x_2,\ldots , y_1,y_2,\ldots \in E^{+}$, $\sum _n x_n \le x, \sum _n y_n \le y$, $\sum _{n\in \mathbb {N}}\Vert x_n\Vert \ge p(x) - \varepsilon$, $\sum _{n\in \mathbb {N}}\Vert y_n\Vert \ge p(y) - \varepsilon$. Considering the sequence $x_1,y_1,x_2,y_2,\ldots$ we find $\sum _{n\in \mathbb {N}}(\Vert x_n\Vert +\Vert y_n\Vert ) \le p(x+y)$. Hence $p(x+y) \ge p(x) + p(y)$.
- On the other hand: Let $z_1,z_2,\ldots \in E^{+}$, $\sum _n z_n \le x+y$; we prove $\sum _{n\in \mathbb {N}}\Vert z_n\Vert \le p(x) + p(y)$. Define $u_n,v_n$ by
  4 $$\begin{aligned} u_1 + \cdots + u_n = (z_1 + \cdots + z_n) \wedge x, \quad v_n= z_n - u_n \quad (n\in \mathbb {N}). \end{aligned}$$
  Then $(z_1+\cdots + z_n) \wedge x - z_n = (z_1 + \cdots + z_n - z_n) \wedge (x- z_n) \le (z_1 + \cdots + z_{n-1}) \wedge x$, implying $u_n - z_n \le 0$; and $(z_1+ \cdots + z_n) \wedge x \ge (z_1+ \cdots + z_{n-1}) \wedge x$, implying $u_n \ge 0$. Thus
  5 $$\begin{aligned} u_n \ge 0, v_n \ge 0 \quad (n\in \mathbb {N}), \end{aligned}$$
  $\sum _{n\in \mathbb {N}}\Vert u_n\Vert \le \sum _{n\in \mathbb {N}}\Vert z_n\Vert <\infty$, so $\sum _n u_n$ exists; $\sum _n u_n \le x$, and $\sum _{n\in \mathbb {N}}\Vert u_n\Vert \le p(x)$. $\sum _{n\in \mathbb {N}}\Vert v_n\Vert \le \sum _{n\in \mathbb {N}}\Vert z_n\Vert <\infty$, so $\sum _n v_n$ exists. For every $n\in \mathbb {N}$, $z_1+ \cdots + z_n \le (z_1+ \cdots +z_n + y) \wedge (x+ y) = (z_1 + \cdots + z_n) \wedge x + y = u_1 + \cdots + u_n + y$, so $v_1+\cdots + v_n \le y$; then $\sum _n v_n \le y$ and $\sum _{n\in \mathbb {N}}\Vert v_n\Vert \le p(y)$. Thus $\sum _{n\in \mathbb {N}}\Vert z_n\Vert \le \sum _{n\in \mathbb {N}}\Vert u_n\Vert + \sum _{n\in \mathbb {N}}\Vert v_n\Vert \le p(x) + p(y)$.$\square$

## Footnotes

1 This follows from the following fact: Let $A,B\subset E$. If *A* and *B* have suprema (infima) in *E*, then so does $A+B$ and $\sup (A+B)= \sup A + \sup B \quad (\inf (A+B)=\inf A + \inf B )$.

2 One could also define the vertical extension in case *E*, $\Omega$, $\Gamma \subset \Omega$ are partially ordered sets (not necessarily vector spaces) and $\varphi : \Gamma \rightarrow E$ is an order preserving map.

3 In some places, e.g., Birkhoff [7], the term ‘integrally closed’ is used.

4 For the construction of the lateral extension, one does not need to assume that $\Gamma$ is directed. However, as one can see later on in the construction, the only part of $\Gamma$ that matters for the extension is $\Gamma ^{+} - \Gamma ^{+}$.

5 Note that for this inclusion it is necessary that $\Gamma$ be directed.

6 By the definition of ideal in [9] or [10] (note that $\Gamma _V$ is directed) this means that $\Gamma _V$ is the smallest ideal in $\Gamma _{LV}$ (and for (b); in $\Gamma _{VL}$) that contains $\Gamma$.

7 A subspace *D* of a partially ordered vector space *E* is called *order dense* in *E* if $x= \sup \{ d\in D: d\le x\}$ (and thus $x= \inf \{d\in D: d\ge x\}$) for all $x\in E$.

8 Our use of the term is similar to the one of van Gaans and Kalauch in [12, Definition 2.3].
